# Shikonin inhibited glycolysis and sensitized cisplatin treatment in non-small cell lung cancer cells via the exosomal pyruvate kinase M2 pathway

**DOI:** 10.1080/21655979.2022.2086378

**Published:** 2022-06-15

**Authors:** Yitian Dai, Yuping Liu, Jingyi Li, Mingming Jin, Hao Yang, Gang Huang

**Affiliations:** a Graduate School, Shanghai University of Traditional Chinese Medicine, Shanghai, China; b Shanghai Key Laboratory of Molecular Imaging, Jiading District Central Hospital Affiliated Shanghai University of Medicine and Health Sciences, Shanghai, China; c Beijing University of Chinese Medicine, Beijing, China; d Qiqihar Medical University, Qiqihar Heilongjiang, China

**Keywords:** Shikonin, non-small cell lung cancer, exosomes, pyruvate kinase M2

## Abstract

The active ingredient of the traditional Chinese medicine comfrey is shikonin, a naphthoquinone compound. The focus of this study was to investigate the effect of shikonin on the proliferation, invasion, migration, and chemoresistance of non-small cell lung cancer (NSCLC) cells, and to explore its underlying molecular biological mechanisms. The results show that shikonin inhibited the viability, proliferation, invasion, and migration of NSCLC cells A549 and PC9, and induced apoptosis. As the inhibitor of pyruvate kinase M2 (PKM2), a key enzyme in glycolysis, shikonin inhibited glucose uptake and the production of lactate, the final metabolite of aerobic glycolysis. *In vivo* chemotherapeutic assay showed that shikonin reduced the tumor volume and weight in NSCLC mice model and increased the sensitivity to cisplatin chemotherapy. Histoimmunology experiments showed the combination of shikonin and cisplatin downregulated the expression of PKM2 and its transcriptionally regulated downstream gene glucose transporter 1 (Glut1) in tumor tissue. In an assessment of glucose metabolism, micro-PET/CT data showed a combination of shikonin and cisplatin inhibited the fluorodeoxy glucose (^18^F-FDG) uptake into tumor. Since exosomal PKM2 affected the sensitivity to cisplatin in NSCLC cells, we also demonstrated shikonin could inhibit exosome secretion and exosomal PKM2 through the administration of exosomal inhibitor GW4869. Furthermore, shikonin sensitized cisplatin treatment by reducing the extracellular secretion of exosomal PKM2. In conclusion, we suggest that shikonin not only inhibits PKM2 intracellularly but also reduces glycolytic flux and increases cisplatin sensitivity through the exosomal pathway.

## GRAPHICAL ABSTRACT

**Figure:**
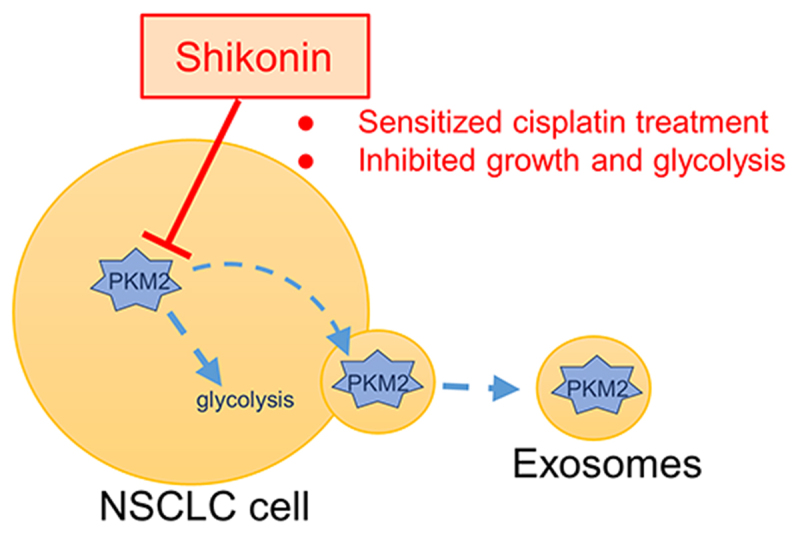

## Highlights

- Shikonin inhibits the proliferation of NSCLC cells by targeting PKM2-mediated glycolysis.
- Shikonin increases the sensitivity of NSCLC cells to cisplatin *in vivo*.
- Shikonin decreases the level of exosomal PKM2 to sensitize cisplatin treatment in NSCLC cells.

## Introduction

Lung cancer accounts for 19.4% of the newly diagnosed tumors worldwide [1]. With the global cancer incidence soaring, China’s situation as a ‘cancer power’ is even less optimistic [2]. The most prevalent (80%) subtype of lung cancer is non-small cell lung cancer (NSCLC). Surgery remains treatment approach for the early stage of NSCLC, with a radical cure rate of less than 35%. Radiotherapy and chemotherapy are often used in the middle and advanced stages, which, although effective for some patients, are limited by considerable side effects, high recurrence and metastasis rates, and poor prognosis. The combination of traditional Chinese medicine formulations and modern chemotherapy drugs can mitigate some of the toxicity associated with the latter and prolong patient survival. Therefore, it is necessary to identify novel therapeutic targets for NSCLC.

Aberrant metabolism is the most important feature of tumor cells, especially aerobic the ‘Warburg effect,’ where in tumor cells get energy primarily through glycolysis even though sufficient oxygen supply [3]. Aerobic glycolysis significantly increases glucose consumption of tumor compared to that of normal cells, which provides the energy required for their high proliferation rates [4,5]. PKM2 mediates the final step of the glycolytic pathway as the most critical rate-limiting enzyme [6] and is highly expressed in breast, lung, rectal, gastric, liver, and other tumor tissues. Increased production of lactic acid, the end product of glycolysis, is extremely related to the occurrence, development, metastasis, and drug resistance of malignant tumors [7]. The altered metabolism of the tumor cells also results in significant changes in the tumor microenvironment that can be potential therapeutic targets [8]. Our recent study showed that [9] hypoxia-induced production of exosomal PKM2 reprogrammed A549 and cancer-associated fibroblasts (CAFs) to create an acidic microenvironment, which in turn promoted the proliferation and cisplatin resistance of NSCLC cells. At the same time, cisplatin-resistant cells could transmit cisplatin resistance to common NSCLC cells through exosomal PKM2.

Comfrey, a traditional Chinese medicine, is the dried root of the comfrey plant found in Xinjiang or Inner Mongolia. It has a sweet/salty taste and ‘cold’ nature that restores heart and liver meridians [10]. Shikonin is a naphthoquinone compound extracted from comfrey, which has antiviral, antioxidant, anti-inflammatory, antitumor, and other effects [11,12]. A study shows [13] that shikonin and its enantiomer inhibited tumor-specific PKM2, and the alkannin is the most specific. However, its mechanism of action in NSCLC is unknown. In this study, we focused on the effects of shikonin on the glycolysis and exosomal pathways of NSCLC cells to analyze its antitumor mechanism. It has opened a new door for the anti-tumor of integrated traditional Chinese and cytotoxic chemotherapy drugs.

In conclusion, we aimed to find high-efficiency and low-toxic active ingredients in natural products for the treatment of tumors. Therefore, a hypothesis is put forward that shikonin is a potential PKM2 inhibitor, by inhibiting the glycolysis and exosomal pathways, ultimately inhibiting the proliferation, invasion, migration, and inducing apoptosis of NSCLC cells.

## Materials and methods

### Cell culture

The water bath at 37°C was preheated to thaw the A549 and PC9 cells (American Type Culture Collection) from the liquid nitrogen tank. The cells were removed from the biosafety cabinet, added to a centrifuge tube containing 5 mL of pre-warmed medium (GIBCO, Grand Island, NY, USA), and centrifuged at 1400 rpm for 3 min at room temperature. Cells were collected, added to cell culture dishes containing 5–10 mL of complete medium, and incubated (37°C, 5% CO_2_). Passage when cells grow to 80–90%.

### Cell viability assay

A549 and PC9 cells were digested with 0.25% trypsin solution, and the cell density was readjusted to 3 × 10^4^cells/mL. The resuspended cells were seeded into 96-well plates at 200 µL/well. The 96-well plate was placed in the incubator for 24 h, and then the medium was changed to an equal volume of shikonin liquid at different concentrations (1 µM, 2 µM, 3 µM, 4 µM, 5 µM, 6 µM, 7 µM, 8 µM, 9 µM, and 10 µM). After 24 h, the drug solution was discarded, and 20 µL of medium and cell counting kit-8 (CCK-8) solution (Bimake, Shanghai, China) were added to each well to mix and dilute at a ratio of 9:1, and were placed in an incubator for cultivation after 2–4 h. The optical density (OD) value of the CCK-8 solution in the 96-well plate was detected by a microplate reader at 450 nm, and the cell-free well was used as a blank control.

### Proliferation assays

The steps for preparing single cell suspension are the same as before. The cells were prepared into a suspension of 1 × 10^4^ cells/mL, and 2 mL of the suspension was equally divided into each well of a 6-well plate, and cultured in a cell incubator for 24 h. The 6-well plate was taken out, the old medium was discarded, and different concentrations of shikonin were added to each group, and cultured in a cell incubator for 10–15 days. During this period, cells were observed every 2–3 days, and the medium was changed in time. When there were obvious clonal plaques on the bottom of the culture dish, the 6-well plate was washed twice with phosphate-buffered saline (PBS), fixed with 4% paraformaldehyde at room temperature for 20–30 min, washed twice with PBS, stained with 0.1% crystal violet for 20–30 min, washed once with PBS, and washed once with double distilled water.

### Transwell assay

The steps for preparing single cell suspension are the same as before. A 24-well plate was prepared with matrigel in a Transwell chamber (Corning Inc, NY, USA), added 200 µL of different concentrations of shikonin containing 1.5 × 10^5^cells to the upper chamber, and added 500 µL of normal medium to the lower chamber after 24 h. The upper and lower chamber medium was then discarded, and the upper chamber surface was gently wiped twice with a cotton swab and washed twice with PBS. They were fixed with 4% paraformaldehyde for 20–30 min, washed twice with PBS, stained with 0.1% crystal violet liquid for 20–30 min, and finally washed twice with PBS. Observation was performed under an inverted fluorescence microscope, and three random fields of view were selected to take pictures and count.

### Wound healing assay

The steps for preparing single cell suspension and plating 6-well plate are the same as before. When the cell density is about 80%, the 6-well plate was streaked and then washed twice with PBS. 2 mL of 6 µM shikonin solution was added to each well and cultured in a cell culture incubator. Using microscope observation, record the scratch width at 0 h, 24 h, and 48 h. The average scratch width was analyzed using ImageJ software.

## Apoptosis assay

The steps of preparing cell suspension, plating 6-well plate, as well as adding drugs are the same as before. Cells were resuspended in 500 µL 1× Binding Buffer, added 5 µL each of fluorescein isothiocyanate (FITC) and propidium iodide (PI) (BD Biosciences, USA) to the above suspension, and protected from light incubate for 15 min at room temperature. 400 µL of 1× Binding Buffer was added to each tube and observed on the machine within 1 h. The experimental results were analyzed and counted using FlowJo software.

### Glucose consumption and lactate production assay

The steps of preparing cell suspension, plating 6-well plate, as well as adding drugs are the same as before. After culturing for 24 h, the old medium was discarded, serum-free Dulbecco’s modified Eagle’s medium (DMEM) was added to each well, and the medium was collected after culturing for 8–12 h [14,15]. Glucose consumption and lactate production levels in the medium were measured using a glucose assay kit (Sigma, USA) and a lactate assay kit (Sigma, USA).

### Western blot analysis

The steps for preparing single cell suspension and plating 6-well plates are the same as before. Briefly, extraction was performed using a radioimmunoprecipitation assay (RIPA) buffer with phenylmethylsulfonyl fluoride (PMSF) and a phosphatase inhibitor cocktail protein. Protein concentration was determined by a protein quantification kit. The proteins were then applied to sodium dodecyl sulfate-polyacrylamide gel electrophoresis (SDS-PAGE) and transferred to polyvinylidene fluoride (PVDF) membranes (Roche, Basel, Switzerland). The membrane was incubated with the primary antibody overnight at 4°C on a destaining shaker, followed by incubation with the secondary antibody for 2 h at room temperature. Finally, protein bands were observed using the Enhanced ECL Chemiluminescent Substrate Kit (Yeasen, Shanghai, China) according to the instructions. The blots were probed with anti-CD63 (1:200; Santa Cruz, sc-5275), anti-PKM2 (1:500; Santa Cruz, sc-365684), and anti-β-actin (1: 500; Santa Cruz, sc-47778) antibodies as per standard protocols.

### Establishment of nude mouse xenograft tumor model

A549 cells were made into a suspension, and the suspension was diluted to 5 × 10^7^ cells/mL with DMEM. A total of 200 µL of the suspension was inoculated subcutaneously on the right side of each nude mouse. After 10–14 days, the tumor-bearing animals were randomly divided into negative control (PBS), low-dose shikonin (0.5 mg/kg/day), high-dose shikonin (2 mg/kg/day), cisplatin (3 mg/kg/day), and combination treatment (2 mg/kg/day shikonin +3 mg/kg/day cisplatin) groups. The respective drugs were administered by intraperitoneal injection once every day. The tumor volume was measured at 3-day intervals, and at the same time, the mice were euthanized at the stipulated time point. The tumor masses were extracted and rinsed with normal saline. The animal experiments were approved (No. 2021-GZR-18-1402225) by the Ethics Committee of Shanghai University of Medicine and Health Sciences.

### Immunofluorescence experiments

The paraffin sections were placed in a 60°C incubator for 20 min, infiltrated with xylene twice, then infiltrated in all levels of alcohol, and finally in distilled water to complete dewaxing. Then, a blocking solution was used, at 37°C incubator for 10 min. The primary antibody dropwise was added at 4°C overnight and then taken out of the refrigerator, returned to room temperature for 10 min, infiltrated three times with PBS, added the secondary antibody dropwise, incubated at 37°C for 30 min, counterstained 2-(4-amidinophenyl)-6-indolecarbamidine dihydrochloride (DAPI) for 2 min, and infiltrated PBS three times. Finally, 75% alcohol for 6 min-85% alcohol for 6 min-absolute ethanol for 6 min-anhydrous ethanol for 6 min-xylene for 15 min was dehydrated and transparent. The paraffin section was taken out, dried a little, and added neutral resin dropwise to the paraffin section for sealing. Fluorescence microscopy was used to observe tissue staining and photographed.

### Immunohistochemistry (IHC) staining experiment

The dewaxing step is the same as before. Then, the paraffin sections were put into a pressure cooker and added with citric acid retrieval solution for antigen retrieval. After 2.5–5 min, they were taken out, cooled to room temperature, and washed three times with PBS. Three percent hydrogen peroxide was incubated at room temperature for 10 min to block endogenous peroxidase and washed three times with PBS. The steps of blocking, adding primary antibody, adding secondary antibody, and developing color are the same as before. Counterstaining, dehydration, and mounting procedures were the same as before, and tissue staining was observed with a microscope and photographed.

### In vivo *^18^F-FDG PET/CT*

The mice were fasted for 6–8 h, weighed, and injected intravenously with 5 MBq ^18^F-FDG contrast agent. The mice were anesthetized with 2% isoflurane 40 min after injection and imaged 5 min later. The maximum standard uptake value (SUV_max_) of the positron emission tomography (PET) images was calculated on the workstation [16].

### Isolation of exosomes

It is divided into five steps, all completed at 4°C. First, about 100 mL of cell culture medium was collected in each group, put into 50 mL centrifuge tubes, and centrifuged at 300 rpm and 2000 rpm for 10 min, respectively. Then, the pellet in the centrifuge tube was discarded, and the cell culture medium after low-speed centrifugation was centrifuged at 10,000 rpm for 30 min. The sediment in the centrifuge tube was discarded again, and the medium after high-speed centrifugation was transferred to a special centrifuge tube for an ultracentrifugation and centrifuged at 100,000 rpm for 90 min (Rotor: Beckman Coulter, SW 32 Ti). Retain the precipitate, at which time the precipitated components are mainly exosomes and some proteins. Finally, the ultracentrifuged pellet was dissolved in PBS and centrifuged at 100,000 rpm for 90 min (Rotor: Beckman Coulter, SW 41 Ti). Discard the supernatant in the centrifuge tube and keep the pellet, which is mainly exosomes at this time [17].

### Transmission electron microscope (TEM)

Taking a 100-mesh sample-loading copper mesh, 10 µL of the exosome sample was dropped on the copper mesh, and placed at room temperature for 1 min. The filter paper was used to absorb the floating liquid, and 10 µL of 3% sodium phosphotungstate solution was added dropwise to the copper mesh with a dropper, and placed at room temperature for 1 min [18]. The filter paper was used to absorb the floating liquid, and the copper mesh was placed under a transmission electron microscope (JEM-1010, Tokyo, Japan) to observe the morphology and take pictures.

### Nanoparticle tracking analysis (NTA)

The exosome concentration was adjusted to 10 µM/mL by resuspending in pre-chilled PBS, followed by 100, 200, 500-fold dilution with PBS. One-milliliter syringe was used to draw the above exosome samples of different concentrations and slowly pushed into the sample chamber. It should be noted that at least 10 different fields of view are analyzed for each video [19]. The detected data were finally analyzed using the analysis software NTA (version 2.3, Thery and Witwer, 2018).

### Statistical analysis

All data in this experiment were carried out in at least three experiments, and the average value was added and subtracted with standard deviation. Significance was determined by comparison between groups. Finally, after the comparison plots were performed using GraphPad Prism 9.0 (Software, USA), P ≤ 0.05 was deemed to be significant.

## Results

This study explored whether shikonin regulates aerobic glycolysis by regulating the expression of PKM2 in NSCLC cells and exosomes, and increases the sensitivity of NSCLC cells to cisplatin. It aims to achieve the purpose of anti-cancer. The results show that shikonin inhibits the viability, proliferation, invasion, and migration, and induces apoptosis of NSCLC cells, in a glycolysis-inhibition manner. Moreover, shikonin decreases the production of exosomes in NSCLC cells and the expression of PKM2 in exosomes to increase the sensitivity to cisplatin.

## Shikonin inhibited the proliferation, invasion, and migration of NSCLC cells

**Figure 1.**
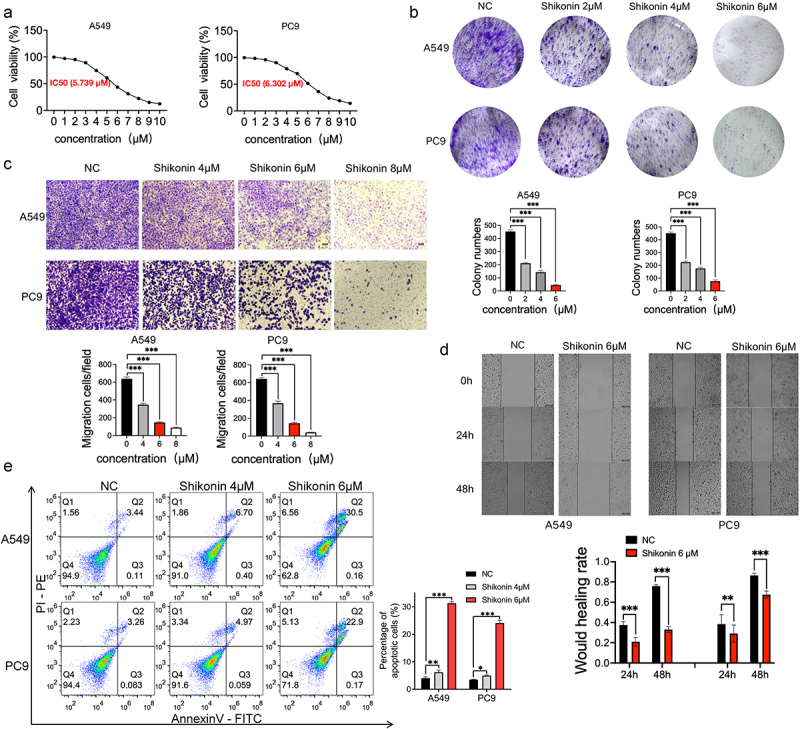
Shikonin suppresses proliferation, invasion and migration of NSCLC cells *in vitro*. (a) Semi-inhibitory concentration of shikonin on concentration gradient treated A549 and PC9 cells. (b) Representative images and number of colonies formed by the control and shikonin-treated A549 and PC9 cells. (c) Representative images of transwell assay and the number of migrating A549 and PC9 cells. (d) Representative images of wound healing assay and the extent of wound coverage in the indicated groups. (e) Flow cytometry plots showing the percentage of apoptotic A549 and PC9 cells treated as indicated. *p < 0.05; **p < 0.01; ***p < 0.001.

The IC_50_ of shikonin against the A549 and PC9 cells at 24 h was 5.739 µM and 6.302 µM, respectively (p < 0.001) (Figure 1a). Therefore, the concentration range of 2 µM, 4 µM, 6 µM, and 8 µM was selected for subsequent experiments. The shikonin-treated cells formed significantly fewer and smaller colonies compared to the blank controls (Figure 1b). In addition, A549 and PC9 cells treated with 4 µM, 6 µM, and 8 µM shikonin showed an obvious decrease in their migration capacity compared to the blank controls in the Transwell assay (Figure 1c). Consistent with this, the extent of wound healing *in vivo* was also weakened after shikonin treatment at 24 h and 48 h (Figure 1d). Finally, shikonin also places a premium on the apoptosis of NSCLC cells in a concentration-dependent manner (Figure 1e). All in all, shikonin can inhibit the malignant potential of NSCLC cells and induce apoptosis.

### Shikonin inhibited glycolysis and PKM2 expression in NSCLC cells

**Figure 2.**
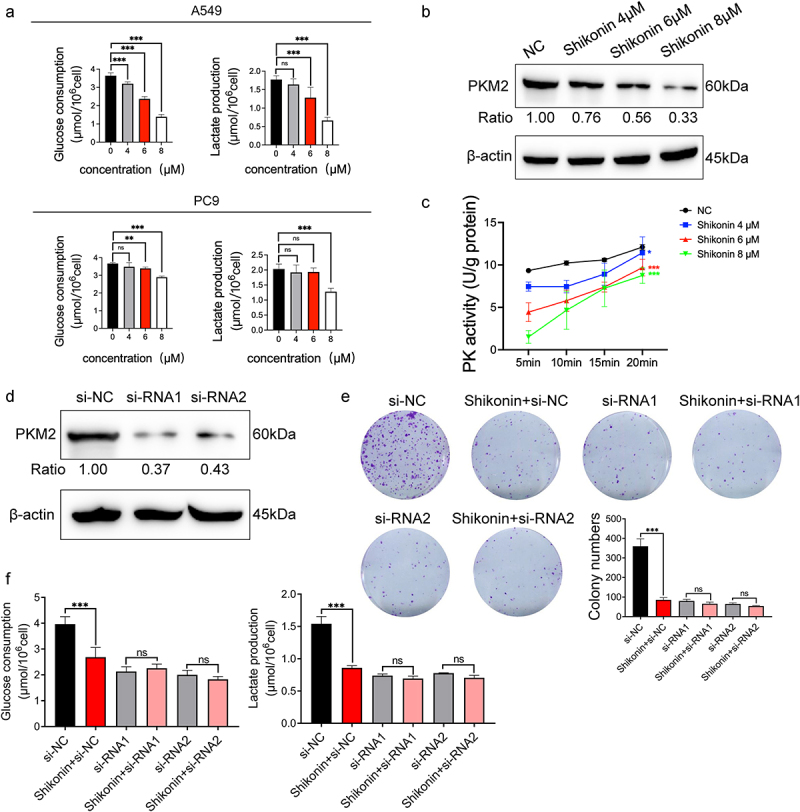
Effects of shikonin on glycolysis and PKM2 expression in NSCLC cells. (a) Glucose uptake and lactate production in A549 and PC9 cells after treatment with 4 µM, 6 µM, and 8 µM shikonin. (b) Immunoblots and relative grayscale values of PKM2 expression in cells treated with 4 µM, 6 µM, and 8 µM shikonin. (c) PKM2 activity in the indicated groups. (d) Immunoblots and relative grayscale values of PKM2 expression in transfected cells. (e) Colonies formed by the control and siRNA transfected cells treated with IC_50_ concentration of shikonin. (f) Changes in glucose uptake and lactate production when shikonin at IC_50_ concentration acts on A549 and siRNA transfected cells. *p < 0.05; **p < 0.01; ***p < 0.001; ns, no significance.

To explore the effect of shikonin on tumor aerobic glycolysis, the A549 and PC9 cells were treated with 4 µM, 6 µM, and 8 µM shikonin, and the glucose uptake and lactate production were measured. Shikonin signaling inhibited glucose uptake and lactate production in A549 cells in a concentration-dependent manner, whereas only high concentrations of shikonin had an effect on the PC9 cells, indicating that A549 cells are more sensitive to the anti-glycolytic effects of shikonin (Figure 2a). For the positive control, 2-deoxy-D-glucose (2-DG), a broad-spectrum inhibitor of glycolysis, had the similar effect of shikonin and significantly inhibited the glycolysis in these cells (Supplementary Figure 1A). Consistent with this, shikonin significantly reduced the expression of (Figure 2b) and activity (Figure 2c) of PKM2 in the A549 cells in a concentration-dependent manner. Although the lactate production decreased, shikonin did not alter the expression of the lactate transporter monocarboxylate transporter 1 (MCT1) and lactate dehydrogenase A (LDHA, Supplementary Figure 1B). To further explore whether the anti-glycolytic effect of shikonin was mediated via PKM2, we knocked down PKM2 in A549 cells by si-RNA1 and si-RNA2 (Figure 2d), and treated them with 6 µM shikonin. Shikonin inhibited the number of colonies formed in A549 cells that normally expressed PKM2. However, shikonin had little effect on colony growth of PKM2-siRNA transfected cells (Figure 2e). Furthermore, although shikonin significantly reduced glucose uptake and lactate production, shikonin was ineffective in PKM2-deficient cells (figure 2f). This suggests that the control of cell growth and glycolysis by shikonin is dependent on the presence of PKM2. To sum up, shikonin inhibits glycolysis in NSCLC cells by inactivating PKM2.

### *Shikonin sensitized NSCLC cells to cisplatin* in vivo

**Figure 3.**
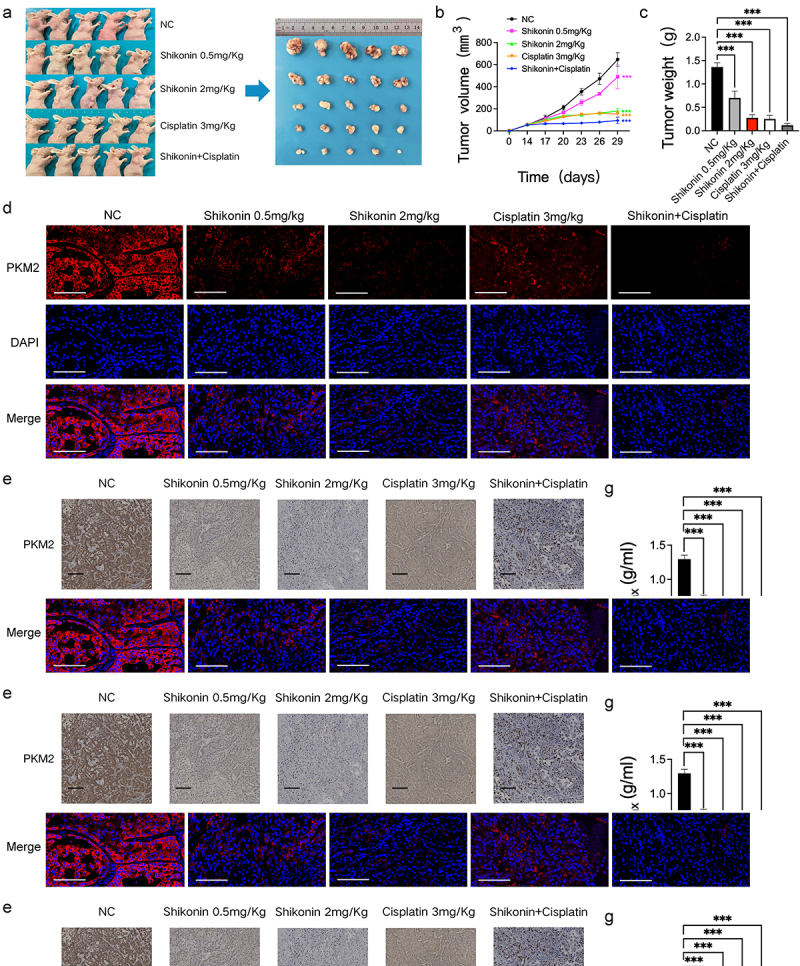
Shikonin affects PKM2-induced cisplatin sensitivity. (a–c) Representative images of tumor-bearing nude mice and representative images of tumor masses in each group, and bar graph showing tumor volume and weight. (d) Representative fluorescence images of tumor tissues showing expression of PKM2 (400×). (e) Representative images of immuno-stained tumor tissues showing the in-situ expression of PKM2 and glut1 (200×). (f–h) ^18^F-FDG micro-PET/CT images of mice of the indicated groups and the SUV_max_ as well as the diameter_max_. **p < 0.01; ***p < 0.001.

A nude mouse subcutaneous tumor model was established to explore the effect of shikonin on cisplatin sensitivity *in vivo* (Figure 3a). The different doses of shikonin and cisplatin significantly reduced tumor volume and weight compared with the blank control group, and the combination of both drugs resulted in maximum therapeutic effect (Figure 3b, 3c and Supplementary Figure 1C). Furthermore, the PKM2 and its transcriptionally regulated downstream gene Glut1 positive rates in the tumor tissues were lower in the shikonin and/or cisplatin-treated groups compared to that in the blank control group (Figure 3d, 3e). PET/CT showed high uptake of ^18^F-FDG in most tumor lesions, and the SUV_max_ of the different treatment groups showed extremely significant differences compared with the blank control group (figure 3f, 3g, 3h). Taken together, shikonin can sensitize NSCLC cells to cisplatin by downregulating PKM2 and reducing glucose uptake by the tumor cells.

### Shikonin decreased exosomal PKM2 to sensitize cisplatin treatment in NSCLC cells

**Figure 4.**
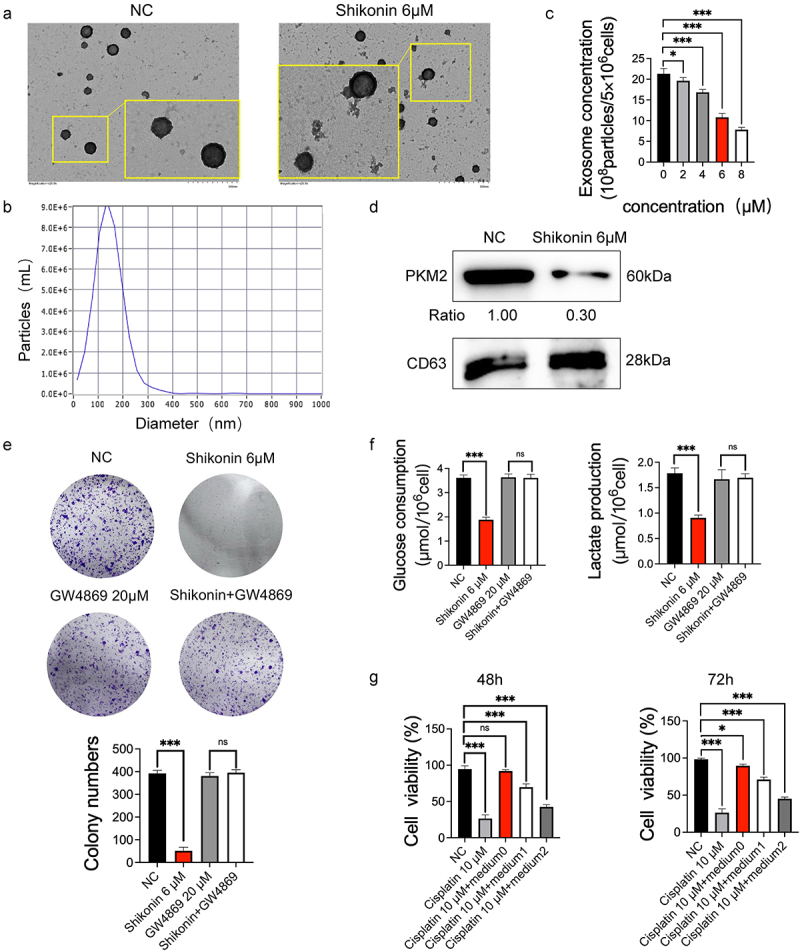
Shikonin decreases exosomal PKM2 to sensitize cisplatin treatment in NSCLC cells. (a–b) Representative TEM images showing exosome morphology and NTA graph showing size distribution. (×25,000) (c) Different concentrations of shikonin act on the relative concentrations of exosomes secreted by A549 cells. (d) Immunoblot and relative grayscale values of PKM2 and the exosomal PKM2 activity in A549 cells with IC_50_ concentration of shikonin. (e) Number of colonies formed by A549 cells with IC_50_ concentration of shikonin with/without GW4869. (f) Glucose uptake and lactate production in A549 cells treated with IC_50_ concentration of shikonin with/without GW4869. (g) Viability of A549 cells cultured in conditioned media from cisplatin-resistant cells in the presence or absence of shikonin (medium0: A549 cisplatin-resistant cells were cultured in serum-free exosome medium for 48 h. medium1, medium2: A549 cisplatin-resistant cells were cultured with shikonin 4 μM and 8 μM for 24 h, respectively, and then replaced with serum-free exosome medium after culturing for 48 h). *p < 0.05; ***p < 0.001; ns, no significance.

Given that PKM2 induces cisplatin sensitivity through exosomal delivery, we wondered whether the anticancer effects of shikonin also involve exosomal PKM2. We successfully isolated the exosomes from A549 and shikonin-treated cells, and confirmed their morphology (Figure 4a) and particle size (Figure 4b) by transmission electron microscopy and NTA, respectively. Shikonin and the positive control 2-DG significantly inhibited the secretion of exosomes from A549 cells concentration-dependent (Figure 4c and Supplementary Figure 1D). Furthermore, treatment with 6 µM shikonin distinctly reduced the expression level of exosomal PKM2 (Figure 4d). Although shikonin reduced colony formation in A549 cells, co-treatment with the exosome inhibitor GW4869 abolished this effect, suggesting that the inhibition of cell proliferation by shikonin is partly dependent on the exosomal pathway (Figure 4e). Similarly, the shikonin-mediated reduction in glucose uptake and lactate production was also rescued by GW4869 (figure 4f). These findings suggest that proliferation and aerobic glycolysis of NSCLC cells were inhibited by shikonin via targeting exosomal PKM2. In a previous study [9], we demonstrated by *in vitro and in vivo* experiments that cisplatin resistance could be transmitted to sensitive NSCLC cells by exosomal PKM2. Consistent with this, cisplatin-sensitive A459 cells cultured with a conditioned medium of cisplatin-resistant cells presented a resistance in the presence of 10 µM cisplatin (Figure 4g). Therefore, cisplatin-resistant NSCLC cells promote a resistance of sensitive cells to cisplatin through exosomes, which can be blocked by shikonin. Shikonin sensitizes cisplatin treatment by reducing the extracellular secretion of exosomal PKM2.

## Discussion

Research on the development of novel anti-cancer drugs is increasingly focusing on plant-derived active compounds given their high efficacy and low toxicity. Comfrey is a medicinal preparation produced in Inner Mongolia and Northeast China, and its main bioactive component is shikonin [20], a naphthoquinone compound with proven effects against hepatocellular carcinoma [21,22], gastric cancer [23] and papillary thyroid cancer [24]. It exerts anti-tumor effects by inducing apoptosis, and inhibiting deoxyribonucleic acid (DNA) topoisomerase and PKM2. Lu et al. [25] reported that shikonin can up-regulate receptor-interacting protein kinase 1 (RIPK1) and receptor-interacting protein kinase 3 (RIPK3) in glioma cells SHG-44, U251, and U87, and increase the production of intracellular reactive oxygen species (ROS). Chen et al. [26] found that shikonin can induce G2/M phase arrest and apoptosis in Ewing sarcoma cells via binding to DNA. Likewise, Wang et al. [27] found that shikonin can induce apoptosis in cisplatin-resistant T24 bladder cancer cells by inhibiting PKM2, which was affected by the RIPK3 inhibitor GSK2399872A (GSK872) or RIP3 si-RNA. Consistent with this, shikonin inhibited the proliferation, migration, invasion, and glycolysis level of NSCLC cells by downregulating PKM2. Furthermore, shikonin also inhibited the secretion of exosomes from the NSCLC cells, as well as the activity of exosomal PKM2. In addition, we found that shikonin exhibited different therapeutic effects on A549 and PC9 cells. Our previously published data also showed that A549 cells treated with resistant cell-derived exosomes remained sensitive at 1 μg/mL cisplatin, whereas transformed PC9 cells were resistant at 1 μg/mL cisplatin [9]. In fact, in addition to cisplatin, A549 and PC9 cells responded differently to multiple drugs, which is in part due to the differential expression of some folate metabolic targets including thymidylate synthase and dihydrofolate reductase [28].

The Warburg effect [29] that refers to the metabolic conversion in tumor cells is mainly aerobic glycolysis to supply enough energy for unlimited proliferation of them. Pyruvate kinase is a key rate-limiting enzyme that catalyzes the final step of glycolysis. PKM2 isoform is highly expressed in tumor cells and plays a crucial role in multiple aspects, such as cell energy supply, cell invasion and metastasis, and epithelial–mesenchymal transition (EMT) [30]. For example, autophagy-related protein 7 (Atg7) and butyrate could inhibit the phosphorylation of PKM2 at tyrosine 105 and reverse the aerobic glycolysis, thereby impeding tumor cell proliferation and EMT [31,32]. The anti-cancer drugs cantharidin, β-elemene, and proanthocyanidin B2 exerted their effects by inhibiting the nuclear translocation of PKM2 [33–36]. Similarly, apigenin downregulated PKM2 in colon cancer cells by inhibiting the Wnt/catenin pathway and reducing the synthesis of the splicing factor polypyrimidine tract-binding protein 1, resulting in decreased cell proliferation [37]. There is increasing evidence that PKM2 activity is associated with tumorigenesis and progression; however, only a handful of studies have focused the attention on exosomes that are also rich in PKM2. Our study showed that shikonin inhibited the proliferation and glycolysis of NSCLC cells by targeting the expression and activity of exosomal PKM2.

Exosomes and their contents regulate tumor cell proliferation, dissemination, and infiltration, and are therefore useful diagnostic, therapeutic, and prognostic markers for cancer [38]. Hannafon et al. [39] found that in lung cancer cells the levels of miRNA-21 and miRNA-1246 were significantly elevated, indicating the diagnostic potential of exosomal miRNAs. Lee et al. [40] found that exosomes containing developmental endothelial locus-1 (DEL-1) and fibronectin detected in the plasma of lung cancer patients can be used as biomarkers for early diagnosis. Furthermore, several anti-cancer drugs can retard tumor progression by inhibiting the secretion of exosomes. For example, the oral antibacterial drug sulfisoxazole specifically inhibited the production and secretion of exosomes from breast cancer cells, and restrained their growth by endothelin receptor A (ETA) [41]. Halofuginone also inhibits exosome secretion from lung cancer cells and downregulates histone deacetylase 2 (HDAC2), which stalls cell cycle transition [42]. In addition, exosomes can also be used as nanocarriers for immunotherapy and gene therapy drugs against lung cancer due to their low immunogenicity.

Current traditional cancer treatment modalities include surgical intervention, radiation therapy, and chemotherapy. However, most lung cancer patients are in the middle or even late stage of lung cancer when they first visit the hospital, which undoubtedly increases the chance of recurrence after treatment [43]. The main reason for this is cisplatin resistance [44]. Although some mechanisms were revealed [45], the cisplatin resistance of NSCLC cells has no effective means and drugs to treat it. This experiment proved for the first time that shikonin inhibits cisplatin-sensitive transmission mediated by exosomal PKM2 in NSCLC cells. This discovery provides a new idea for the synergistic treatment of tumors with traditional Chinese medicine and modern chemotherapeutic drugs. However, this study has certain limitations, such as the lack of sufficient clinical patient samples to demonstrate the effect of shikonin on primary human tumor cells. Furthermore, given the technically challenging methods of exosome isolation and purification, our findings cannot be clinically translated at this juncture.

## Conclusions

Shikonin inhibits the proliferation of NSCLC cells by targeting PKM2-mediated glycolysis and sensitizes the treatment to cisplatin by blocking exosome transmission. Therefore, integrating traditional Chinese medicine and chemotherapy drugs can be a promising new direction in anti-tumor therapy.

## Supplementary Material

Supplemental Material
