# Supplementary material for: Shikonin inhibited glycolysis and sensitized cisplatin treatment in non-small cell lung cancer cells via the exosomal pyruvate kinase M2 pathway
Source: Bioengineered. 2022 Jun 15;13(5):13906–18. doi: 10.1080/21655979.2022.2086378 (PMC9275963; doi:10.1080/21655979.2022.2086378)
Supplement: Supplemental Material [file KBIE_A_2086378_SM9309.zip › supplementary/Ethical approvement.pdf]

上海健康医学院医学伦理审查报告

批件编号	2021-GZR-18-1402225		
审查日期		审查地点	科技处
审查方式	☐ 会议审查　　☐ 快速审查		
研究方案名称	非小细胞肺癌起始细胞来源的小细胞外囊泡携带磷酸化的PKM2 传播化疗耐药性的机制研究		
申报项目类型	国家自然科学基金		
项目负责人	杨浩	职 称	副教授
研究起止时间：	2021.1-2026.12		

涉及动物实验的主要内容：

NSCLC 耐药及其发生机制是亟待研究和解决的问题， sEV 中的 PKM2 具有促使 NSCLC 细胞产生顺铂耐药性的作用。本研究旨在探究 CIC 细胞分泌的 sEV PKM2 在肿瘤耐药传递中的功能及其环境响应性触发条件。在动物实验中，本研究为了揭示 PKM2 Y105 磷酸化的非小细胞肺癌对顺铂化疗耐药性的影响，建立 PKM2 WT 和 Y105F 稳定表达的 CIC 细胞的小鼠皮下荷瘤模型，并同时进行 4 mg/kg 的顺铂静脉注射 2 周，对 Y105 磷酸化在小鼠肿瘤顺铂化疗治疗中的作用进行探究。

学校意见：

伦理委员会对本课题研究方案涉及的动物实验目的、动物分组及使用数量、实验方法、动物处死方法等进行综合评估。研究者充分考虑实验动物的利益，善待动物，尽量防止或减少动物的应激、痛苦和伤害。动物实验目的和方法符合动物福利与伦理标准。同意申报本项目。

上海健康医学院 科研伦理委员会

公章

日期：　　年　　月　　日
