# Supplementary material for: Shikonin inhibited glycolysis and sensitized cisplatin treatment in non-small cell lung cancer cells via the exosomal pyruvate kinase M2 pathway
Source: Bioengineered. 2022 Jun 15;13(5):13906–18. doi: 10.1080/21655979.2022.2086378 (PMC9275963; doi:10.1080/21655979.2022.2086378)
Supplement: Supplemental Material [file KBIE_A_2086378_SM9309.zip › supplementary/Gating strategy for flow cytometry.pptx]

## Slide 1
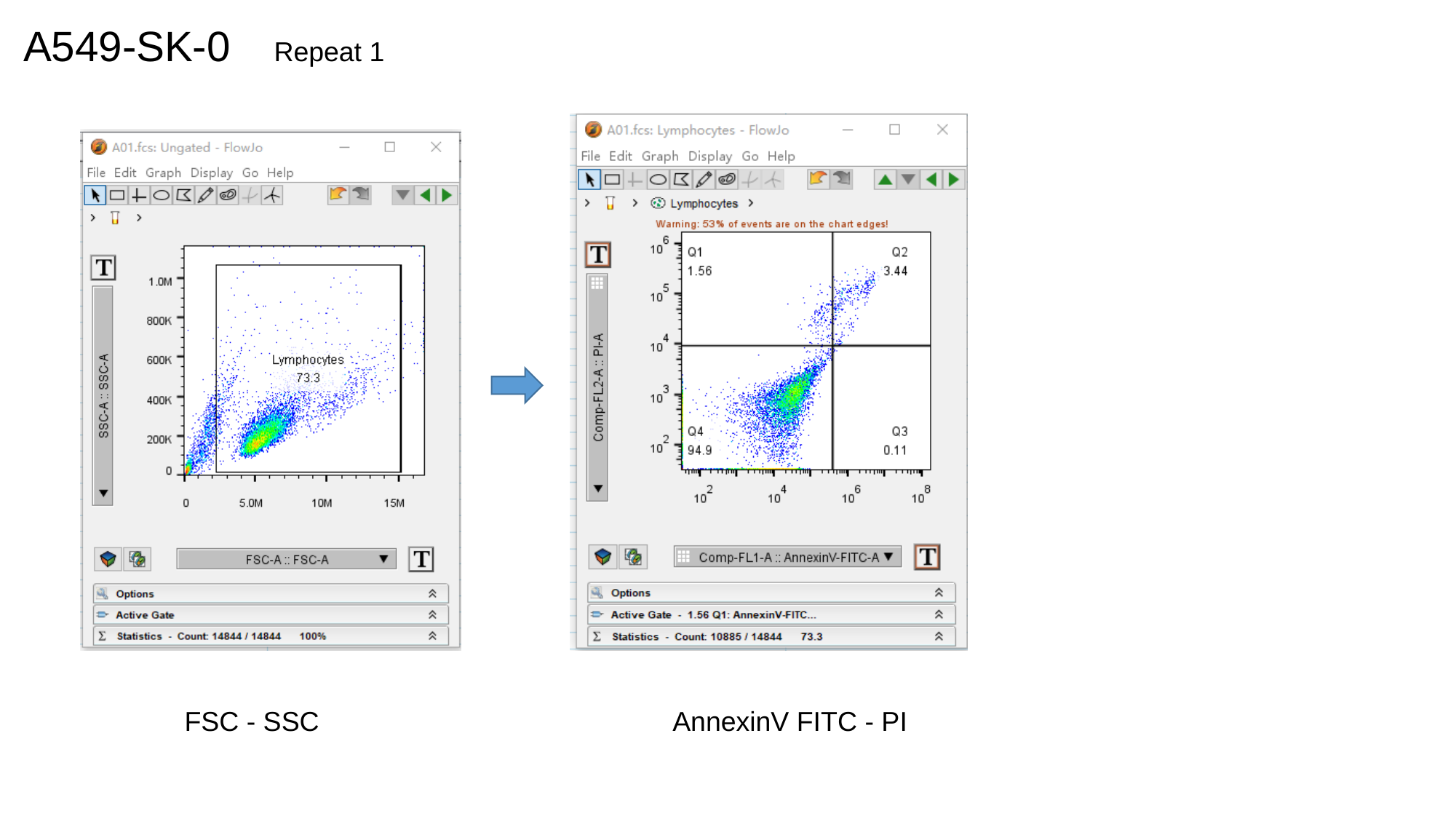

A549-SK-0 Repeat 1
FSC - SSC
AnnexinV FITC - PI

## Slide 2
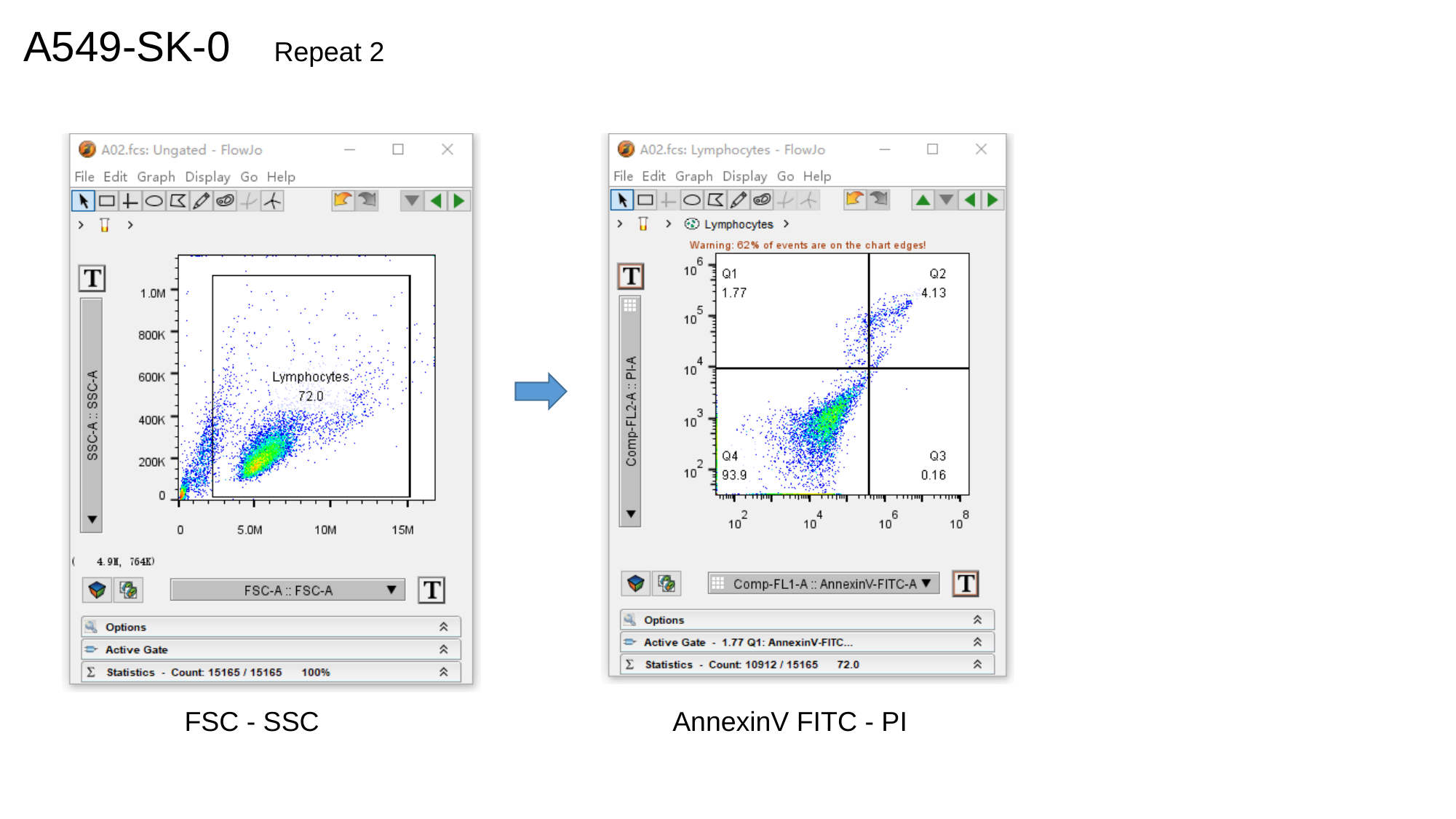

A549-SK-0 Repeat 2
FSC - SSC
AnnexinV FITC - PI

## Slide 3
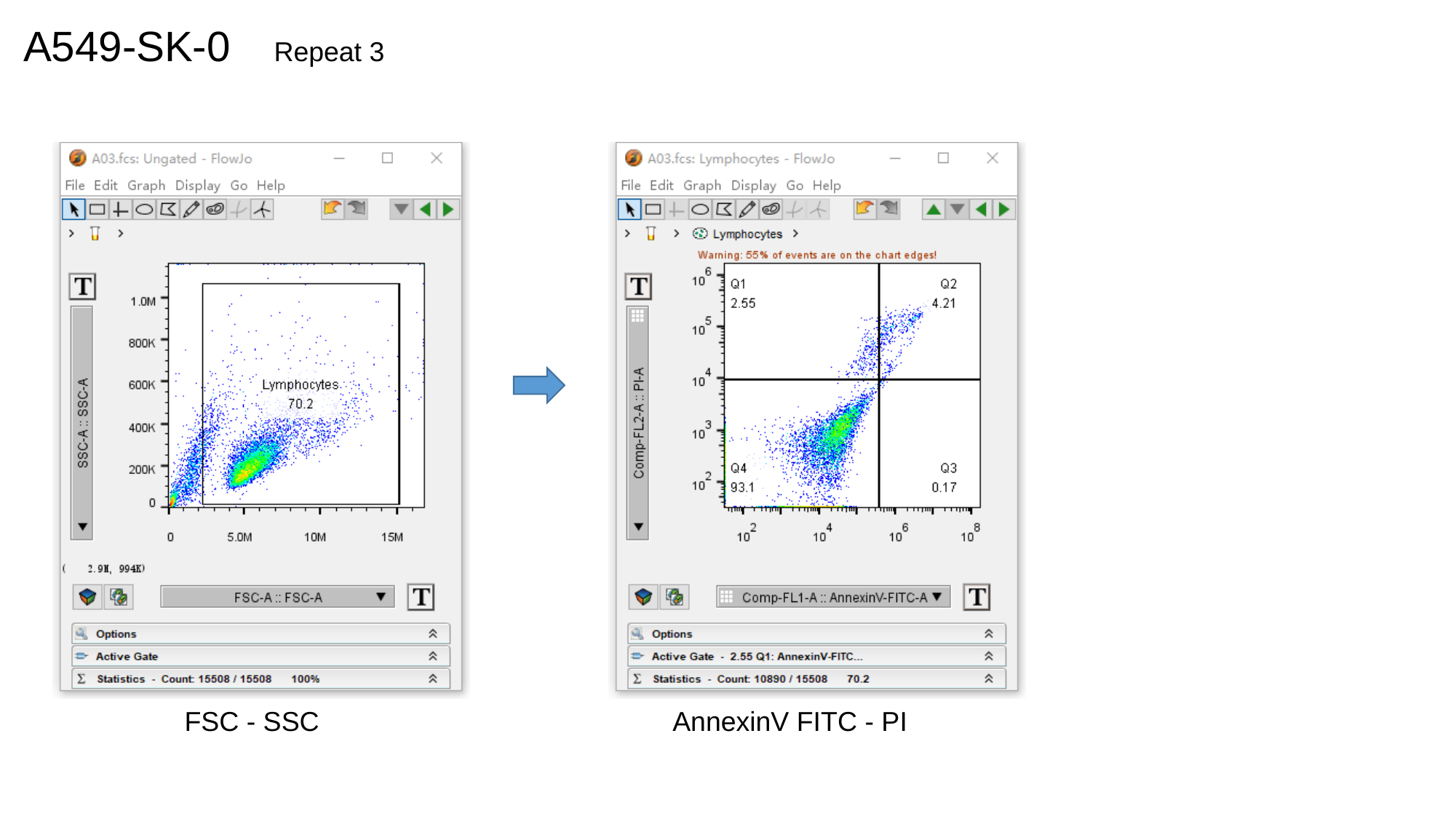

A549-SK-0 Repeat 3
FSC - SSC
AnnexinV FITC - PI

## Slide 4
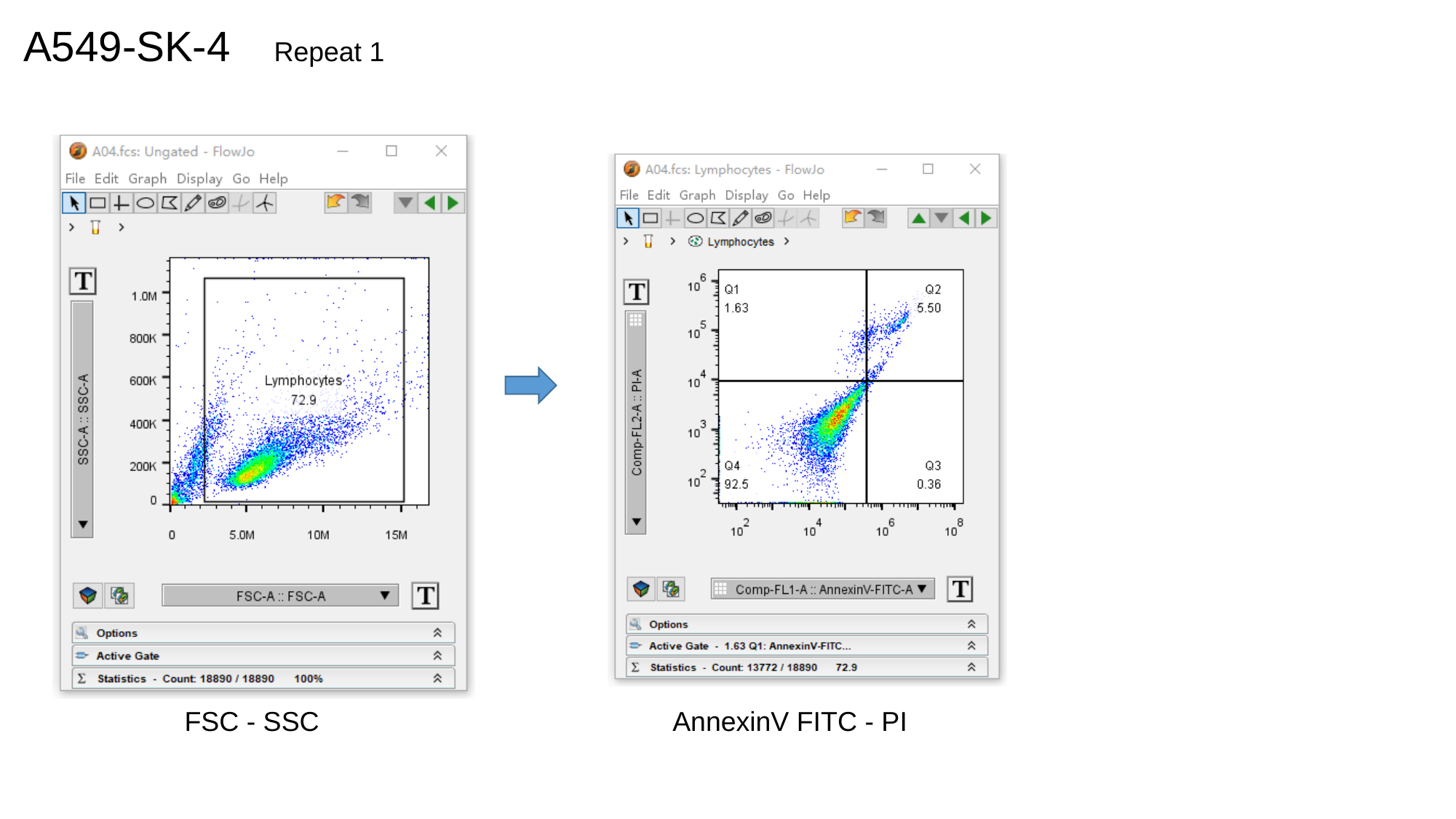

A549-SK-4 Repeat 1
FSC - SSC
AnnexinV FITC - PI

## Slide 5
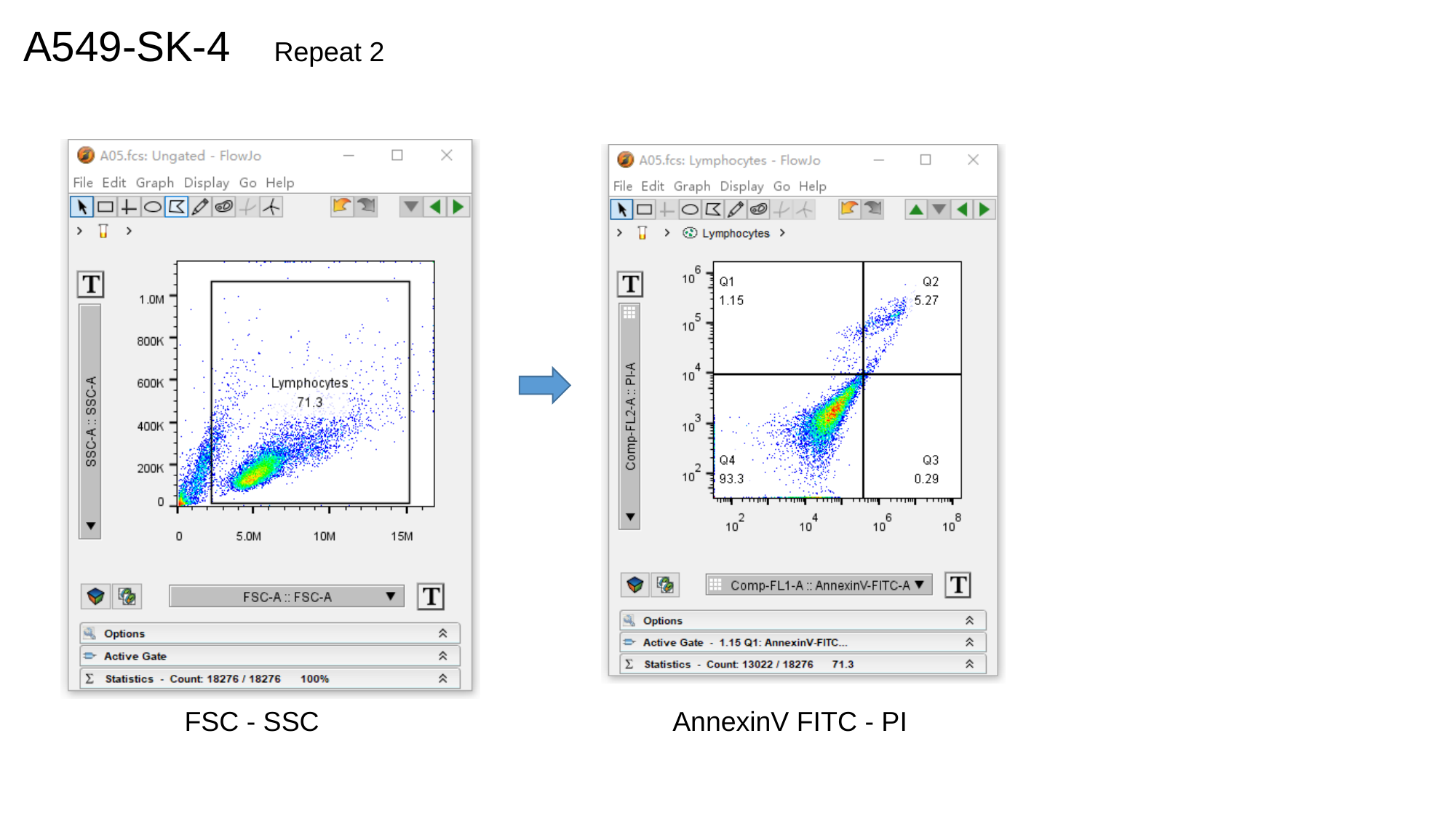

A549-SK-4 Repeat 2
FSC - SSC
AnnexinV FITC - PI

## Slide 6
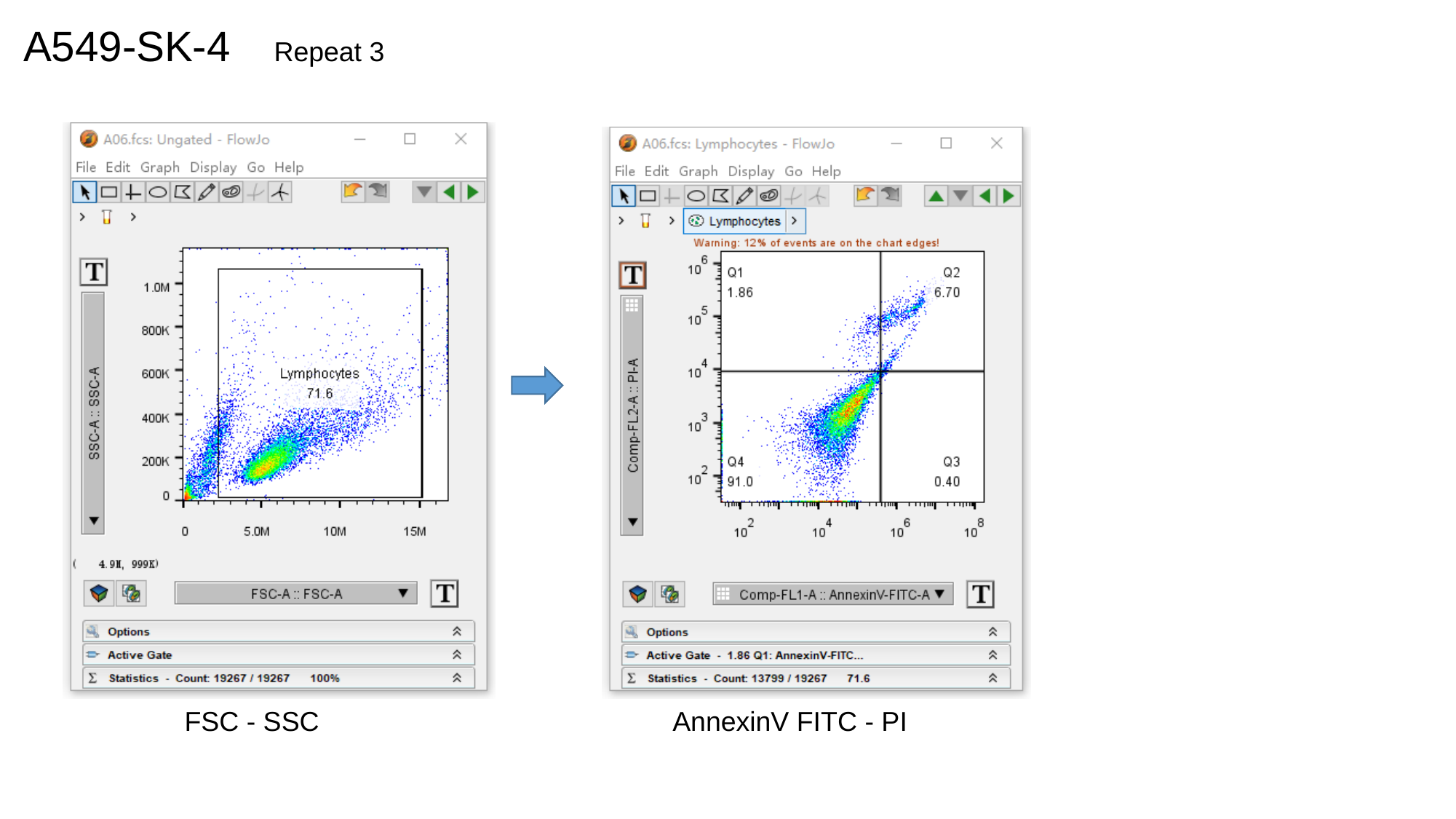

A549-SK-4 Repeat 3
FSC - SSC
AnnexinV FITC - PI

## Slide 7
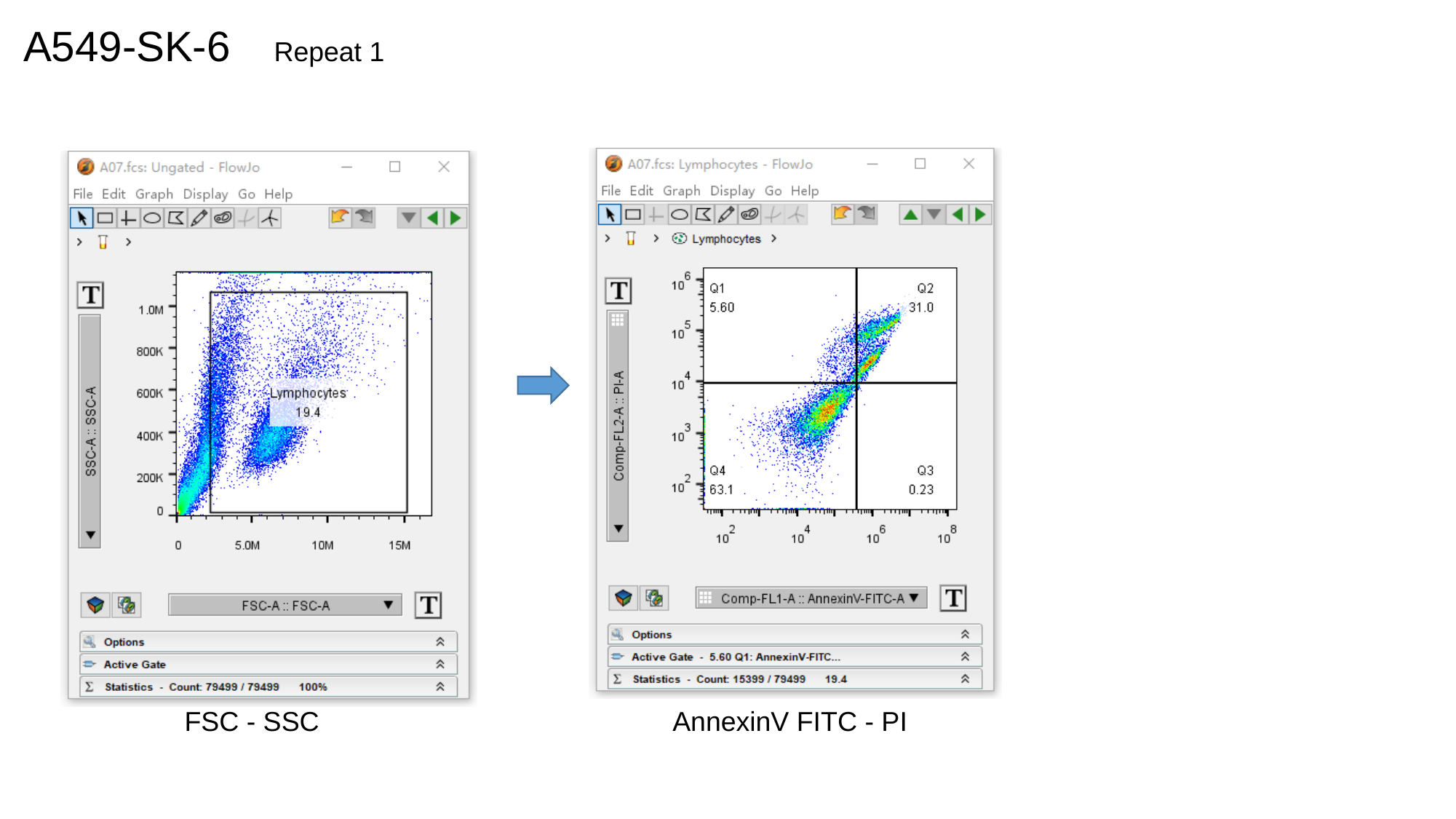

A549-SK-6 Repeat 1
FSC - SSC
AnnexinV FITC - PI

## Slide 8
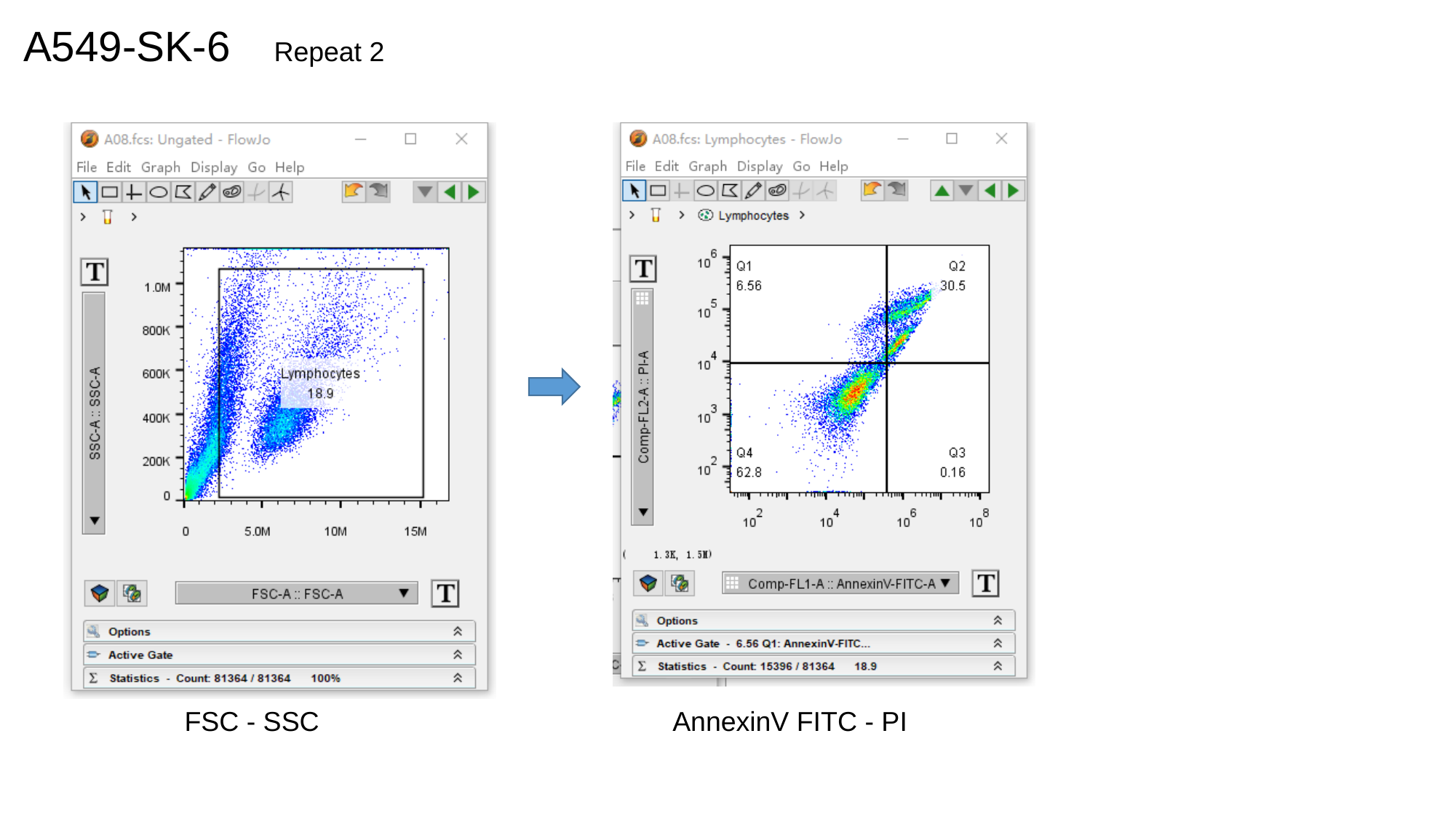

A549-SK-6 Repeat 2
FSC - SSC
AnnexinV FITC - PI

## Slide 9
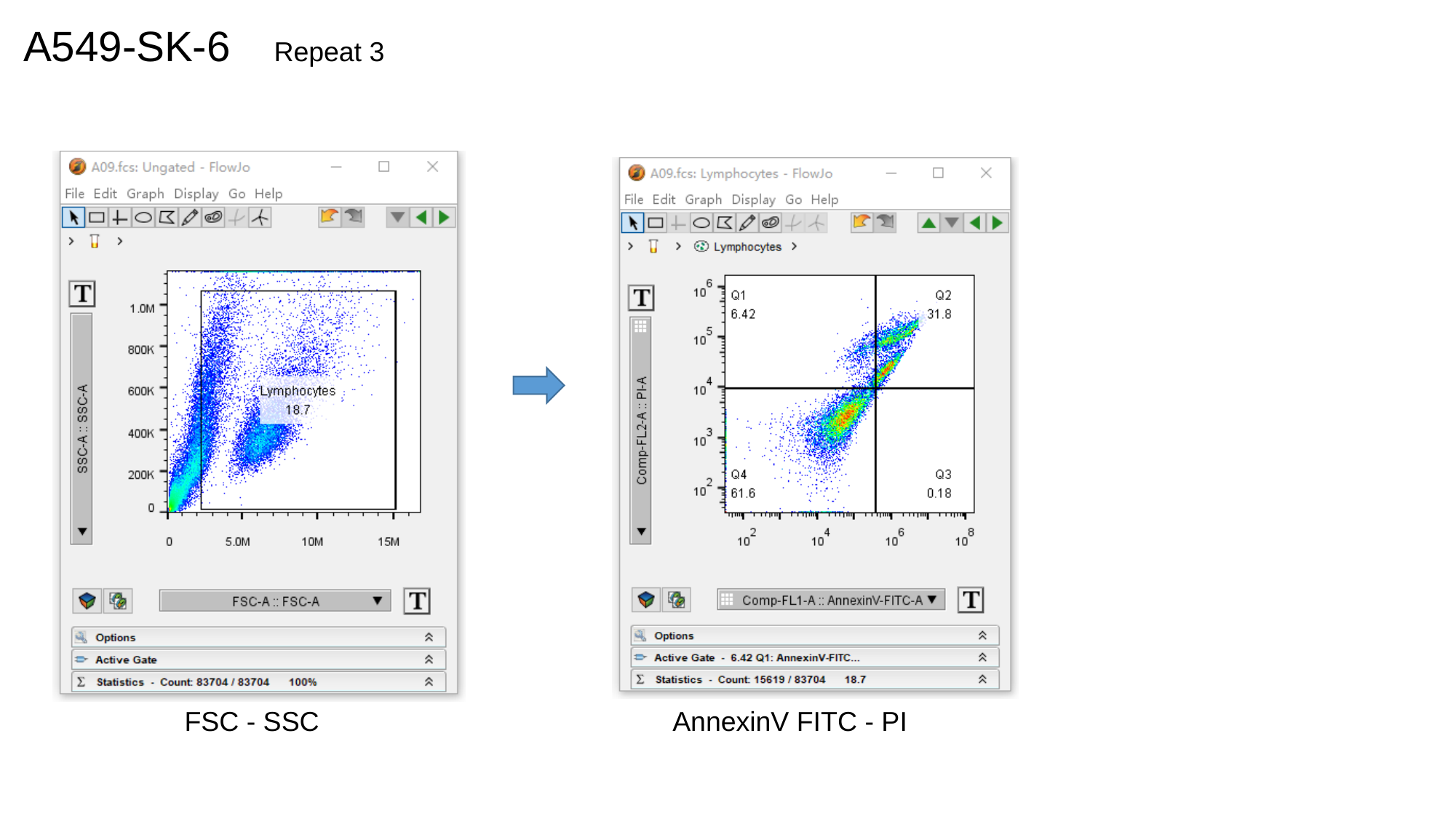

A549-SK-6 Repeat 3
FSC - SSC
AnnexinV FITC - PI

## Slide 10
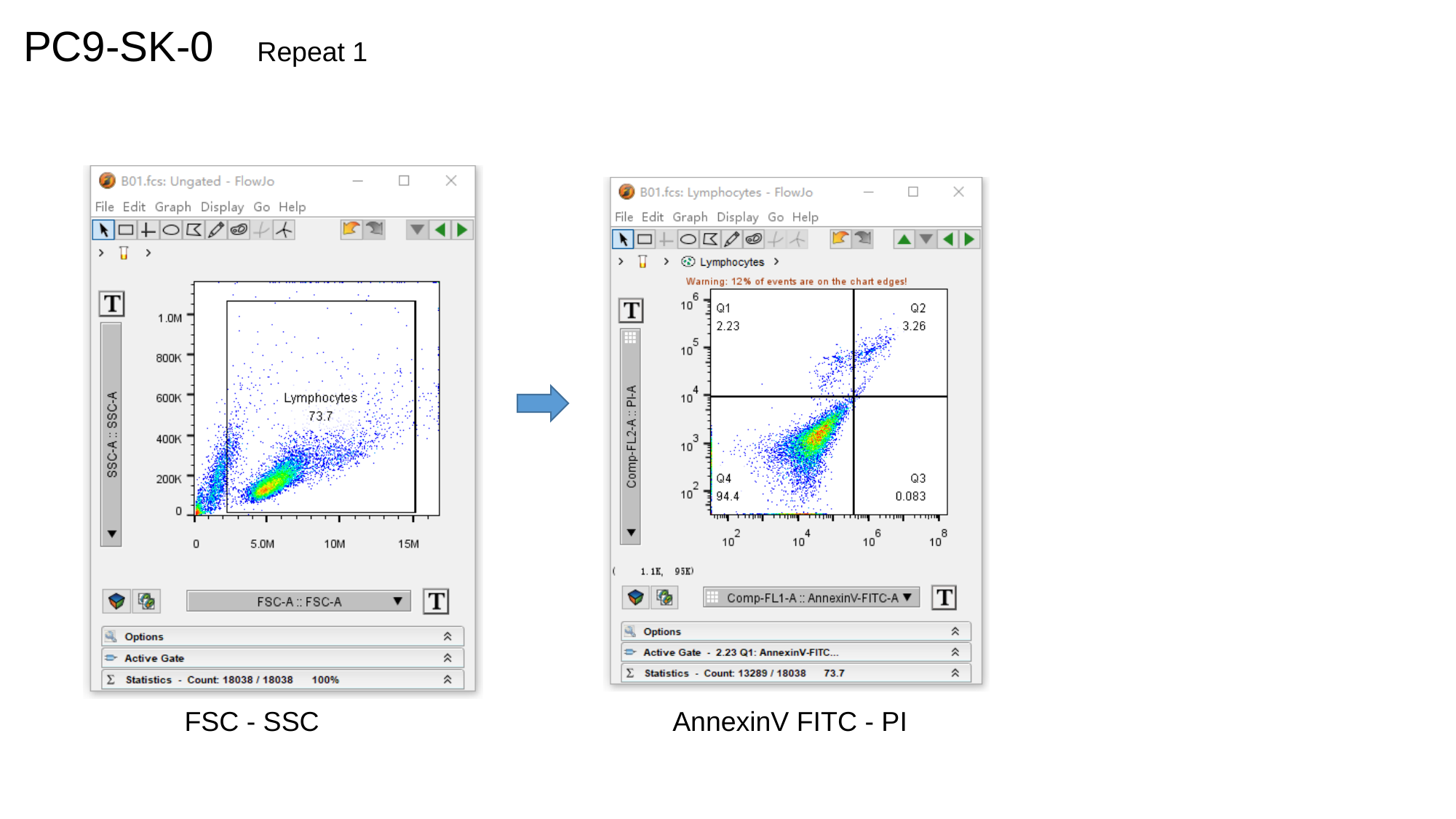

PC9-SK-0 Repeat 1
FSC - SSC
AnnexinV FITC - PI

## Slide 11
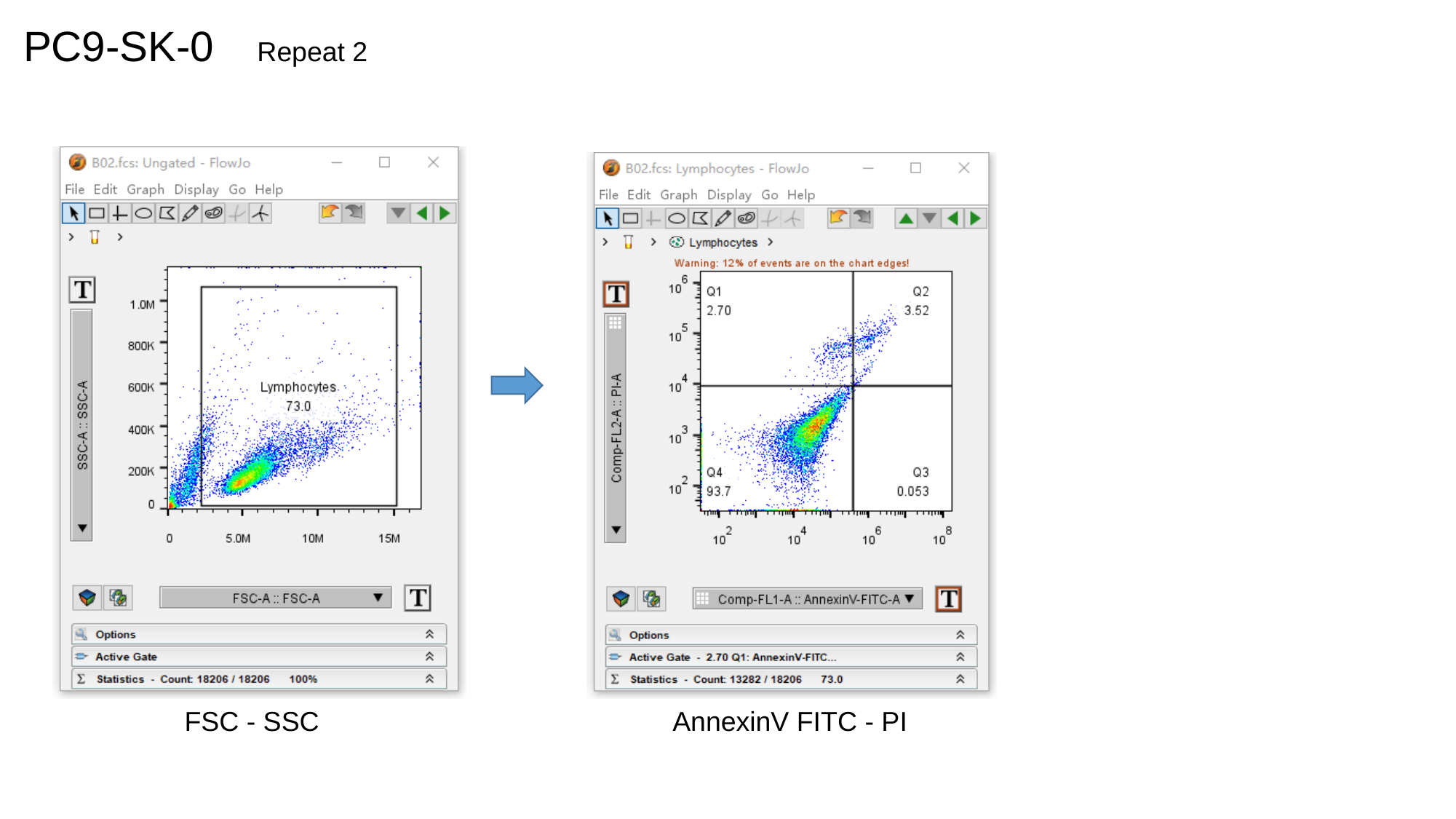

PC9-SK-0 Repeat 2
FSC - SSC
AnnexinV FITC - PI

## Slide 12
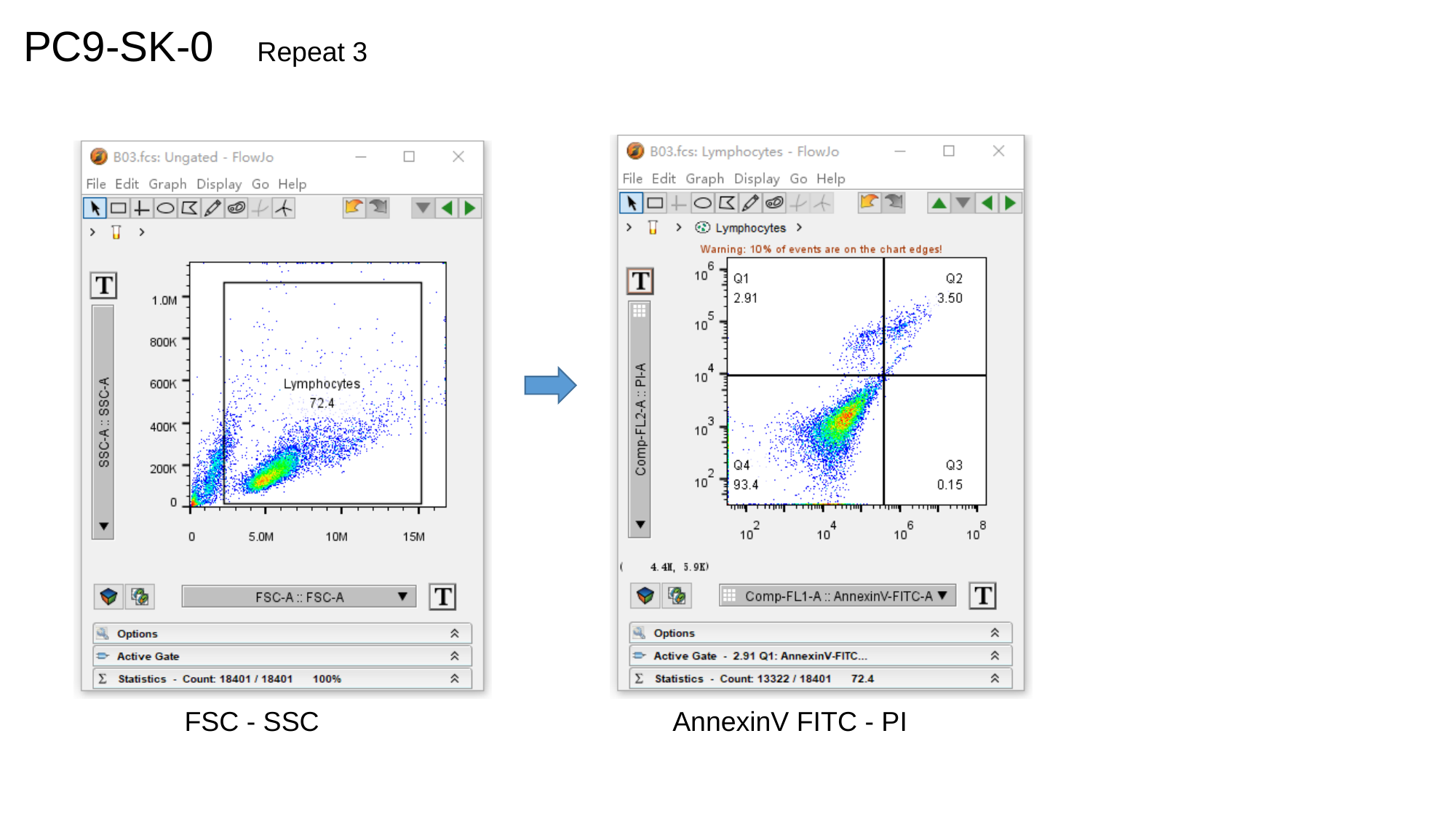

PC9-SK-0 Repeat 3
FSC - SSC
AnnexinV FITC - PI

## Slide 13
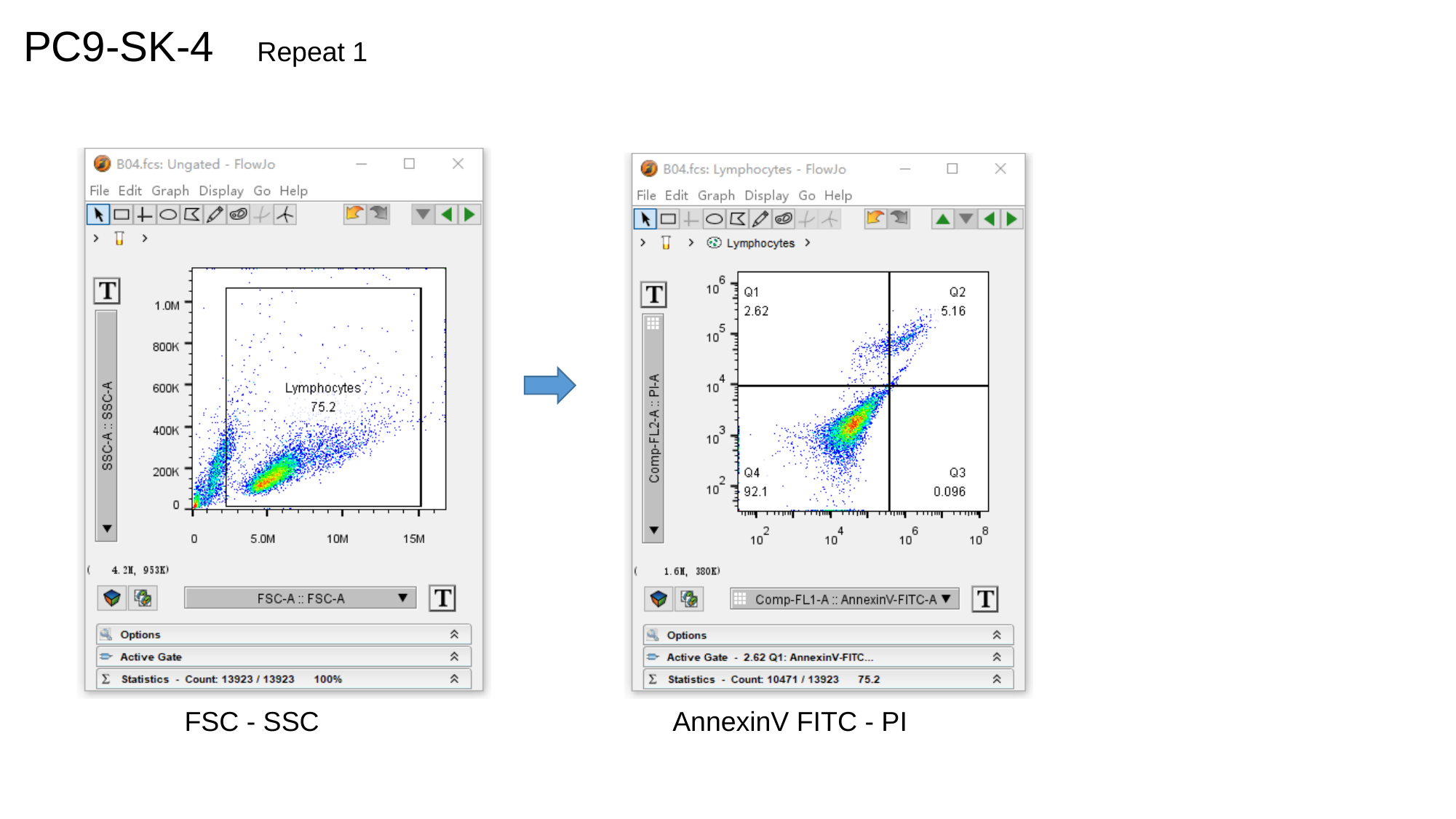

PC9-SK-4 Repeat 1
FSC - SSC
AnnexinV FITC - PI

## Slide 14
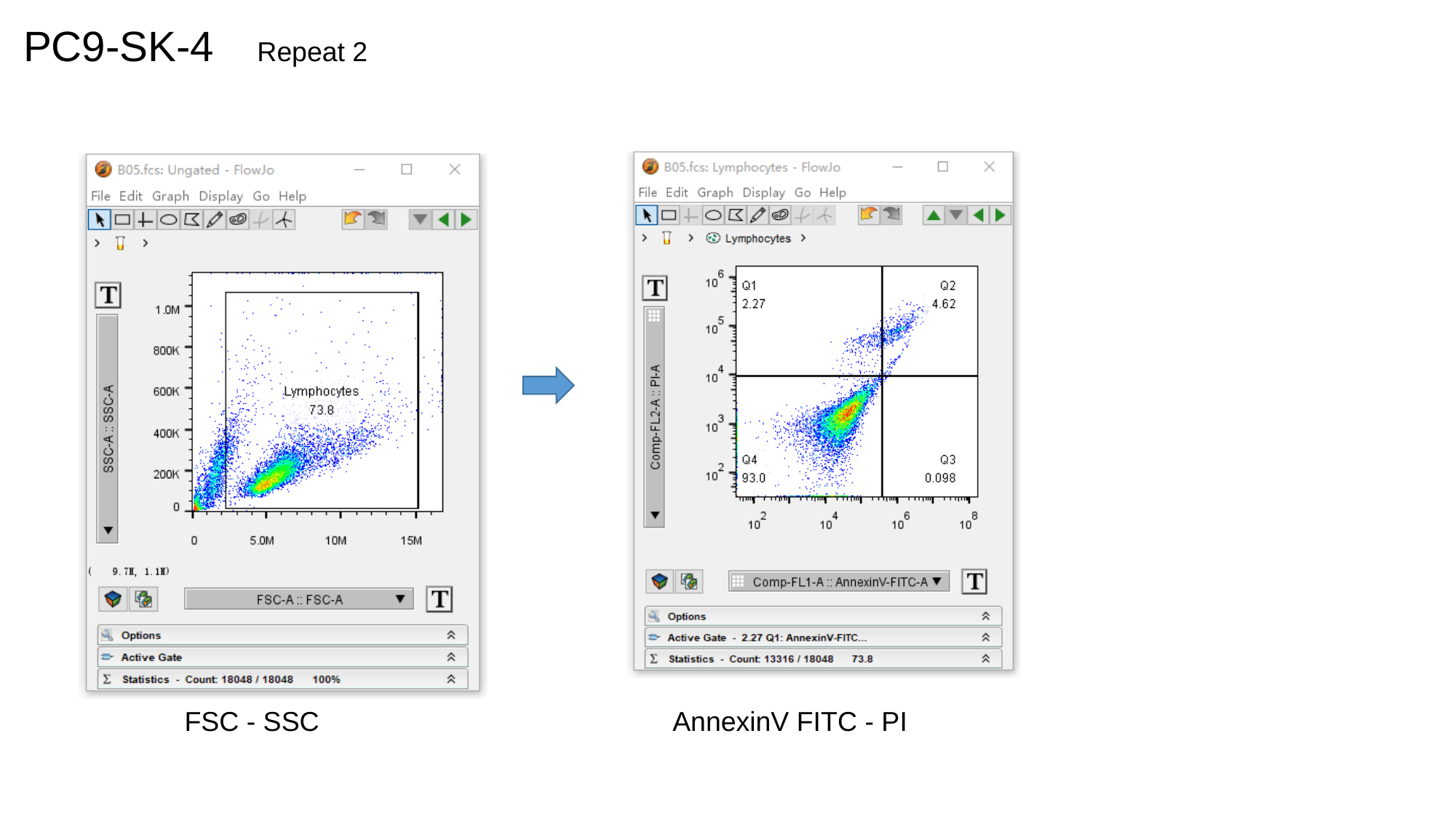

PC9-SK-4 Repeat 2
FSC - SSC
AnnexinV FITC - PI

## Slide 15
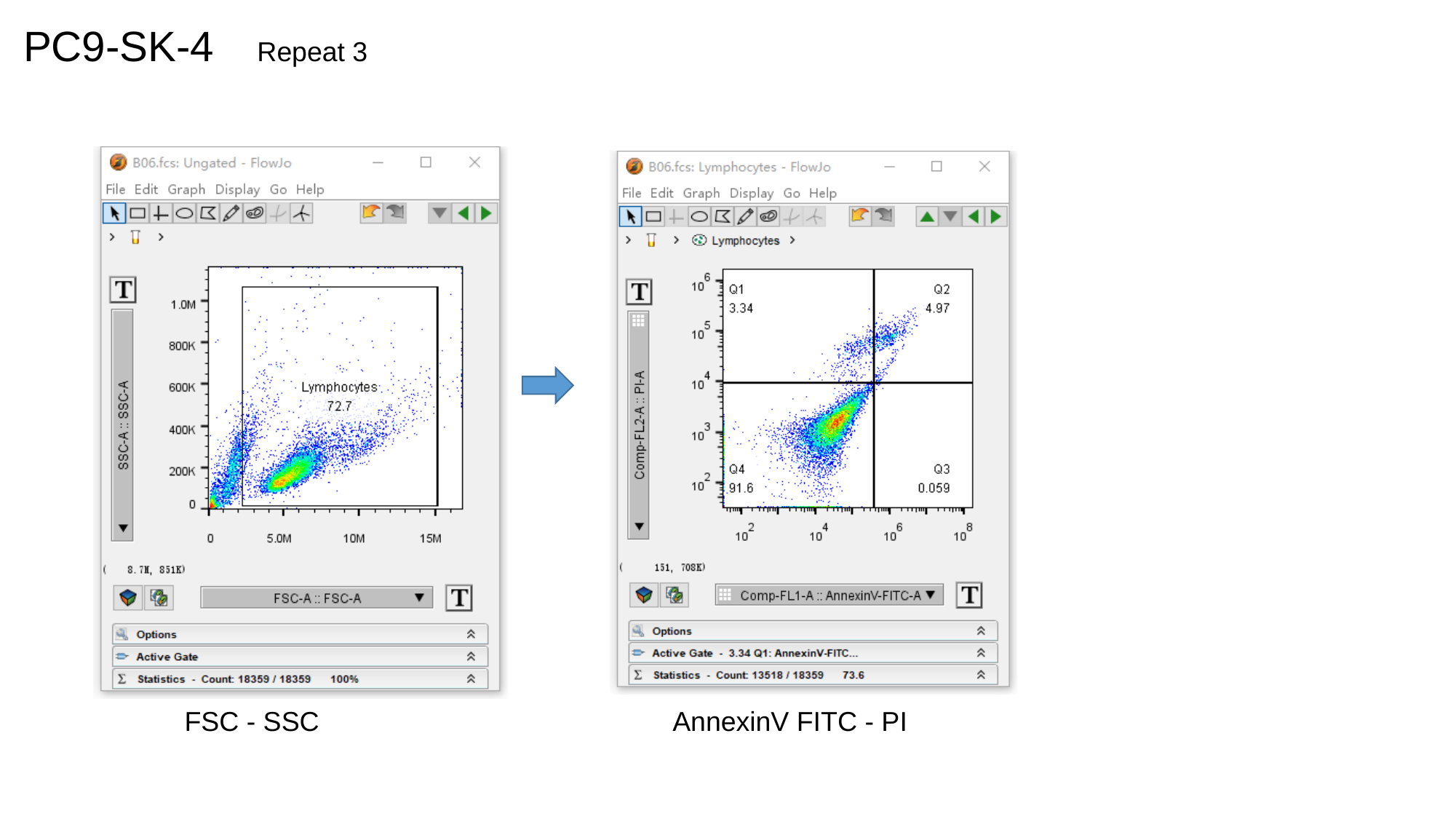

PC9-SK-4 Repeat 3
FSC - SSC
AnnexinV FITC - PI

## Slide 16
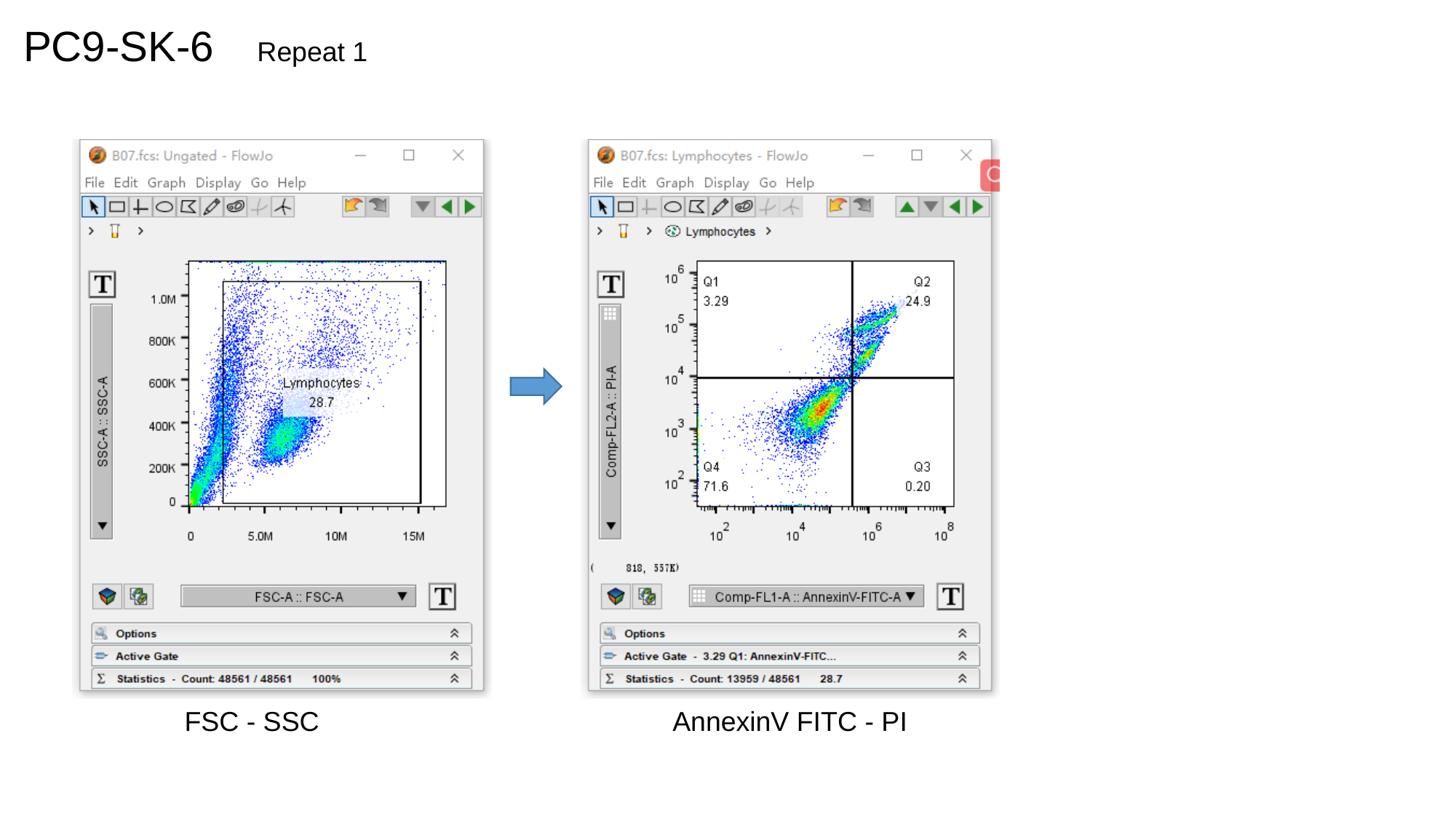

PC9-SK-6 Repeat 1
FSC - SSC
AnnexinV FITC - PI

## Slide 17
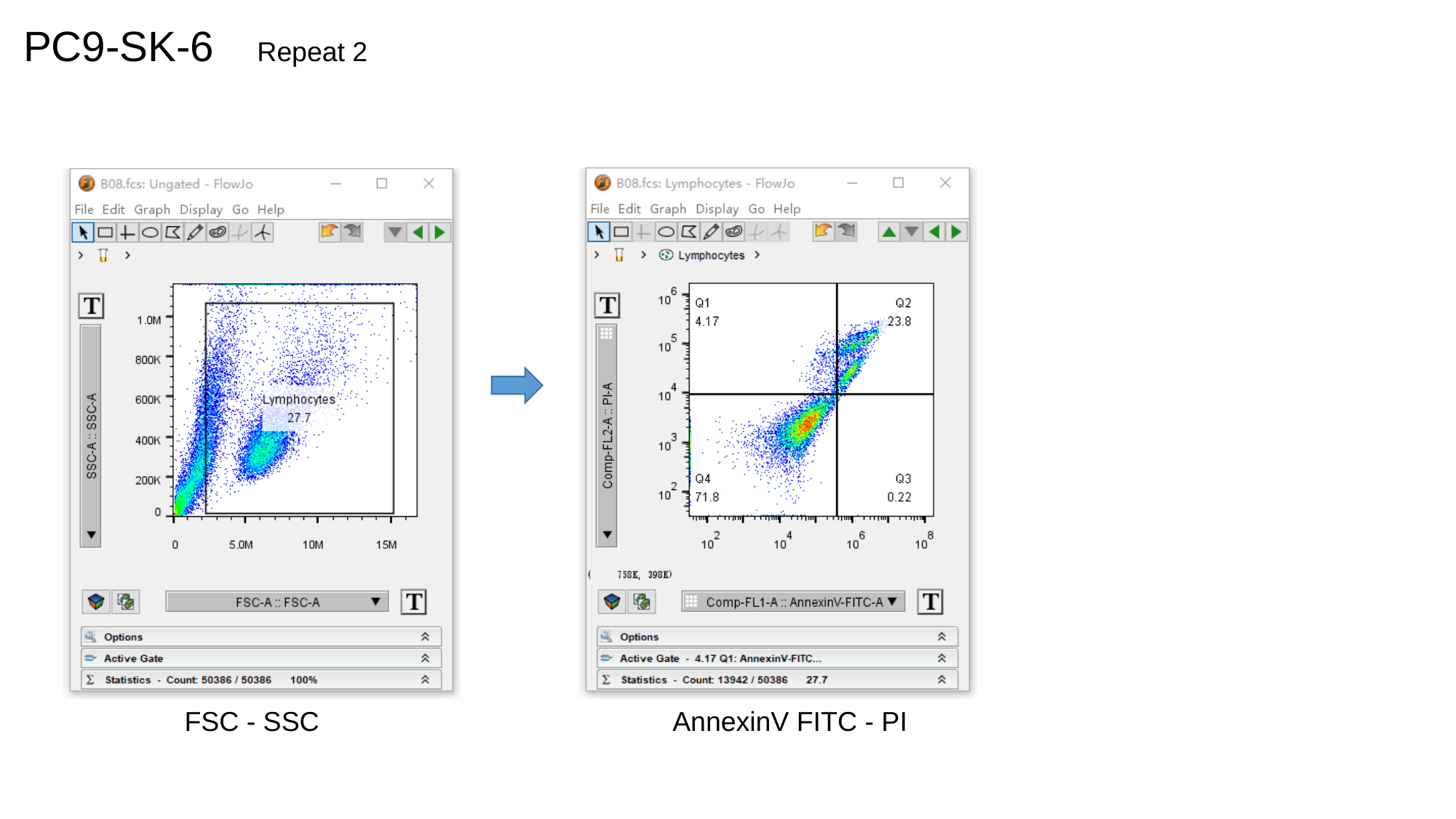

PC9-SK-6 Repeat 2
FSC - SSC
AnnexinV FITC - PI

## Slide 18
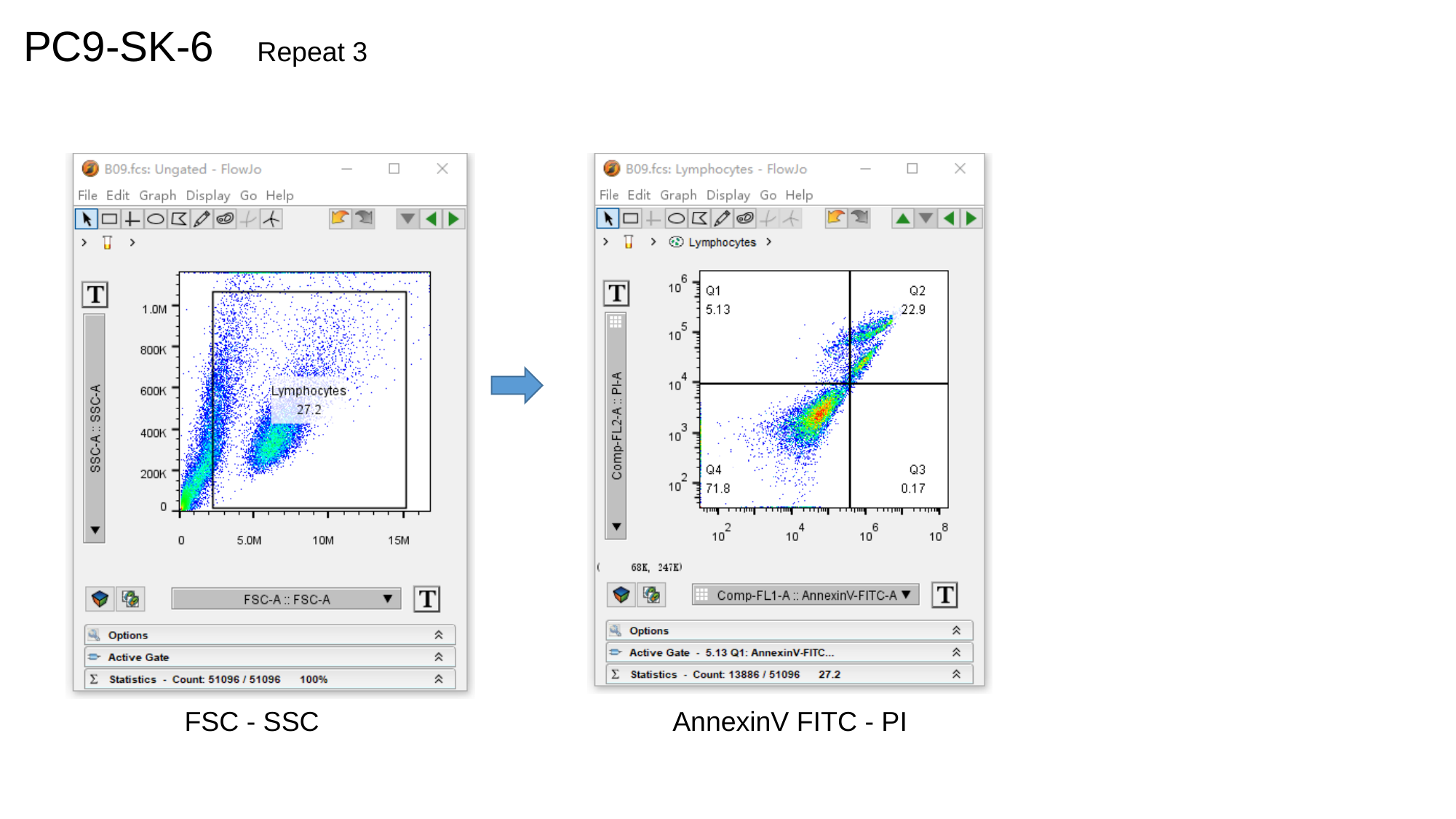

PC9-SK-6 Repeat 3
FSC - SSC
AnnexinV FITC - PI
