# Supplementary material for: Shikonin inhibited glycolysis and sensitized cisplatin treatment in non-small cell lung cancer cells via the exosomal pyruvate kinase M2 pathway
Source: Bioengineered. 2022 Jun 15;13(5):13906–18. doi: 10.1080/21655979.2022.2086378 (PMC9275963; doi:10.1080/21655979.2022.2086378)
Supplement: Supplemental Material [file KBIE_A_2086378_SM9309.zip › supplementary/Original WB and microscopy images.pptx]

## Slide 1
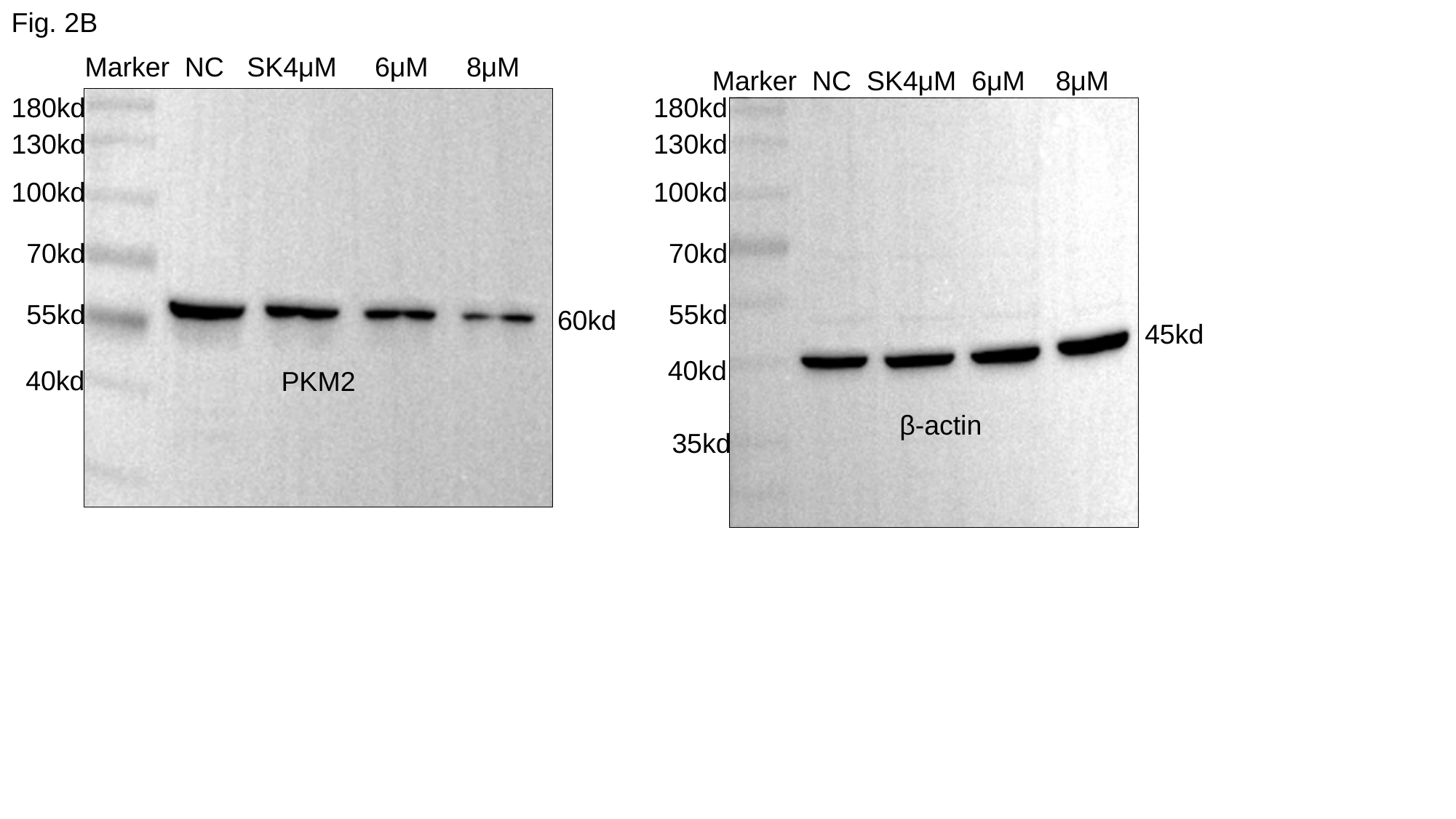

Fig. 2B
Marker NC SK4μM 6μM 8μM
Marker NC SK4μM 6μM 8μM
180kd
180kd
130kd
130kd
100kd
100kd
70kd
70kd
55kd
55kd
60kd
45kd
40kd
40kd
PKM2
β-actin
35kd

## Slide 2
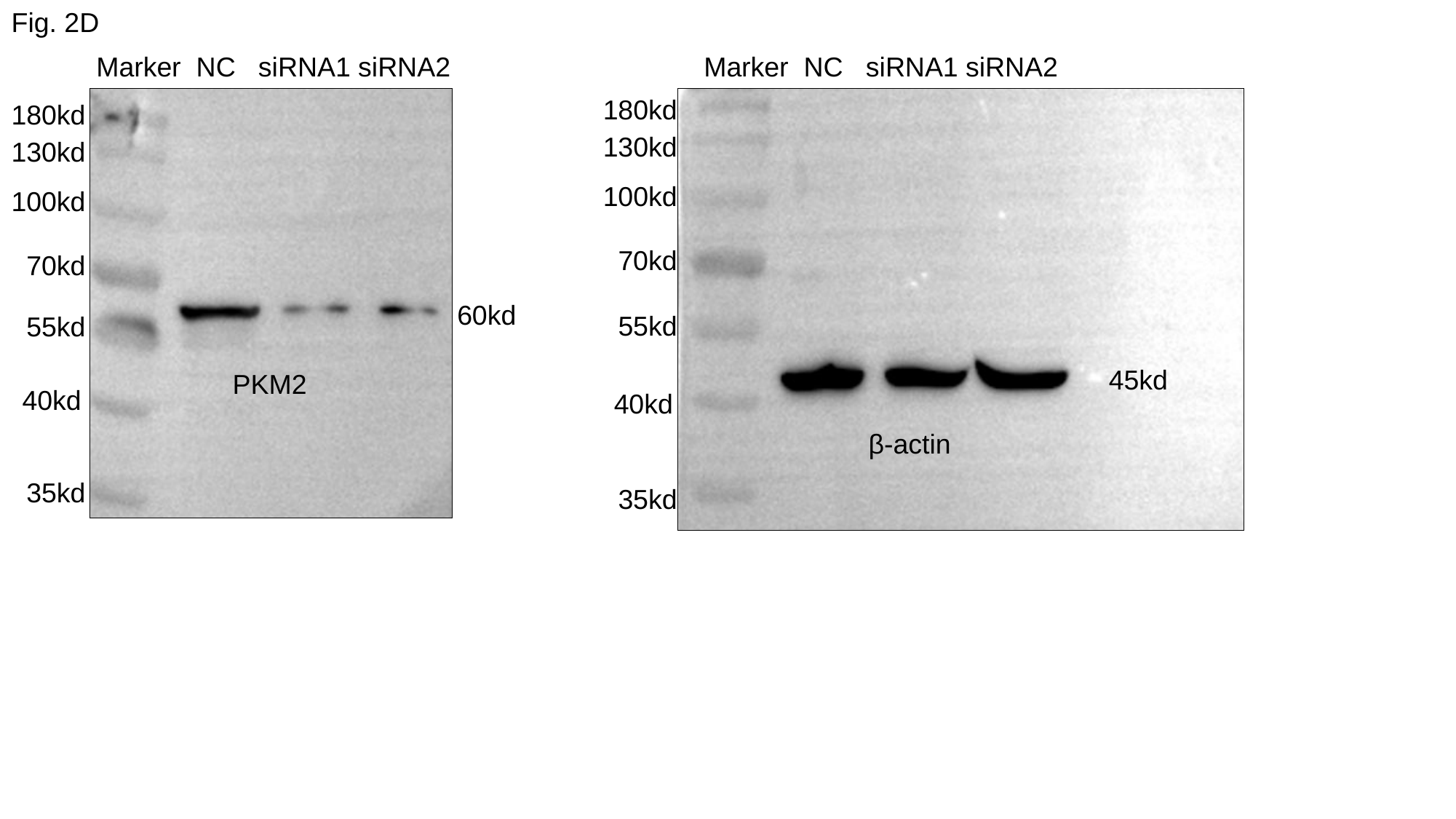

Fig. 2D
Marker NC siRNA1 siRNA2
Marker NC siRNA1 siRNA2
180kd
180kd
130kd
130kd
100kd
100kd
70kd
70kd
60kd
55kd
55kd
45kd
PKM2
40kd
40kd
β-actin
35kd
35kd

## Slide 3
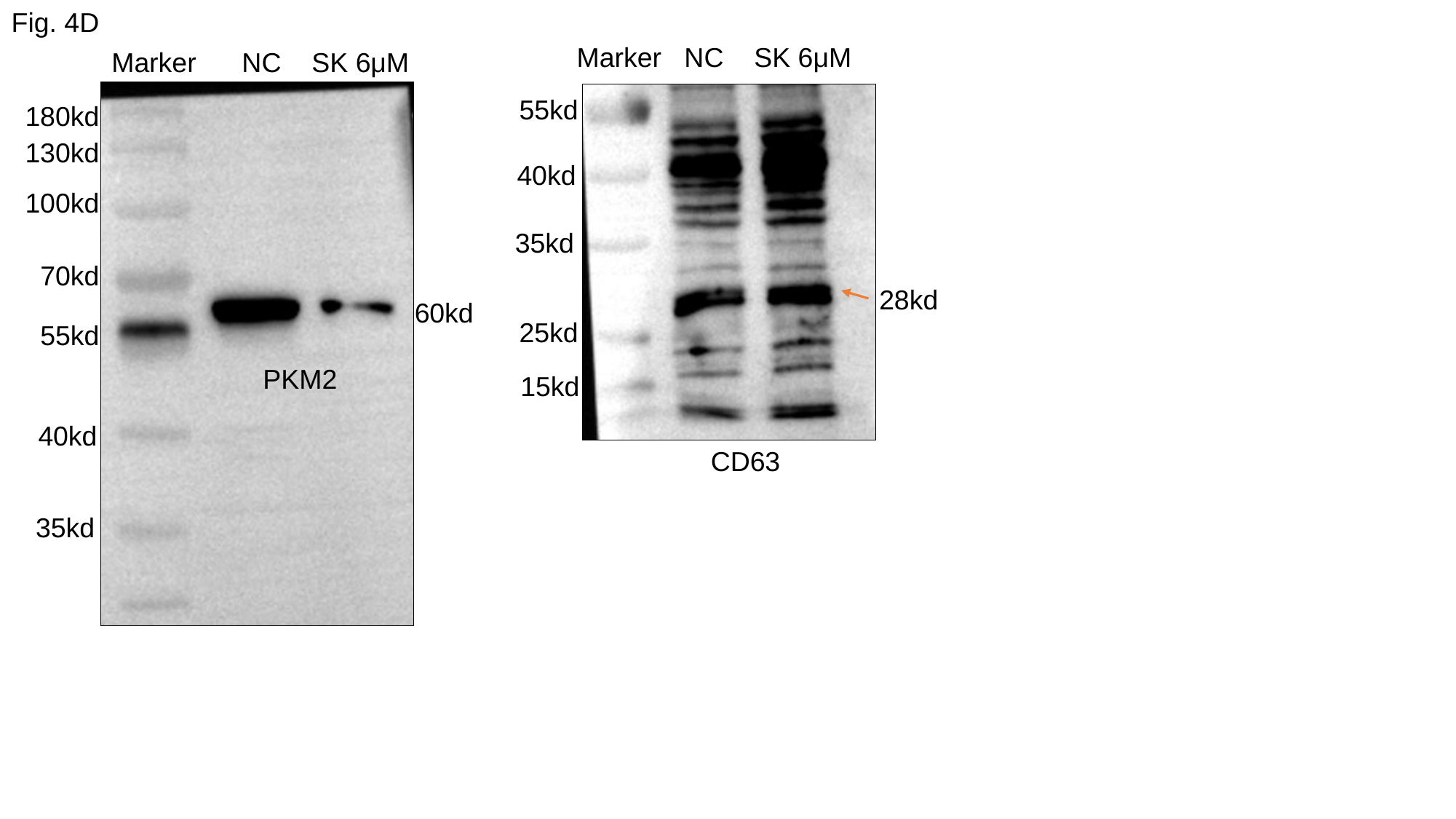

Fig. 4D
Marker NC SK 6μM
Marker NC SK 6μM
55kd
180kd
130kd
40kd
100kd
35kd
70kd
28kd
60kd
25kd
55kd
PKM2
15kd
40kd
CD63
35kd

## Slide 4
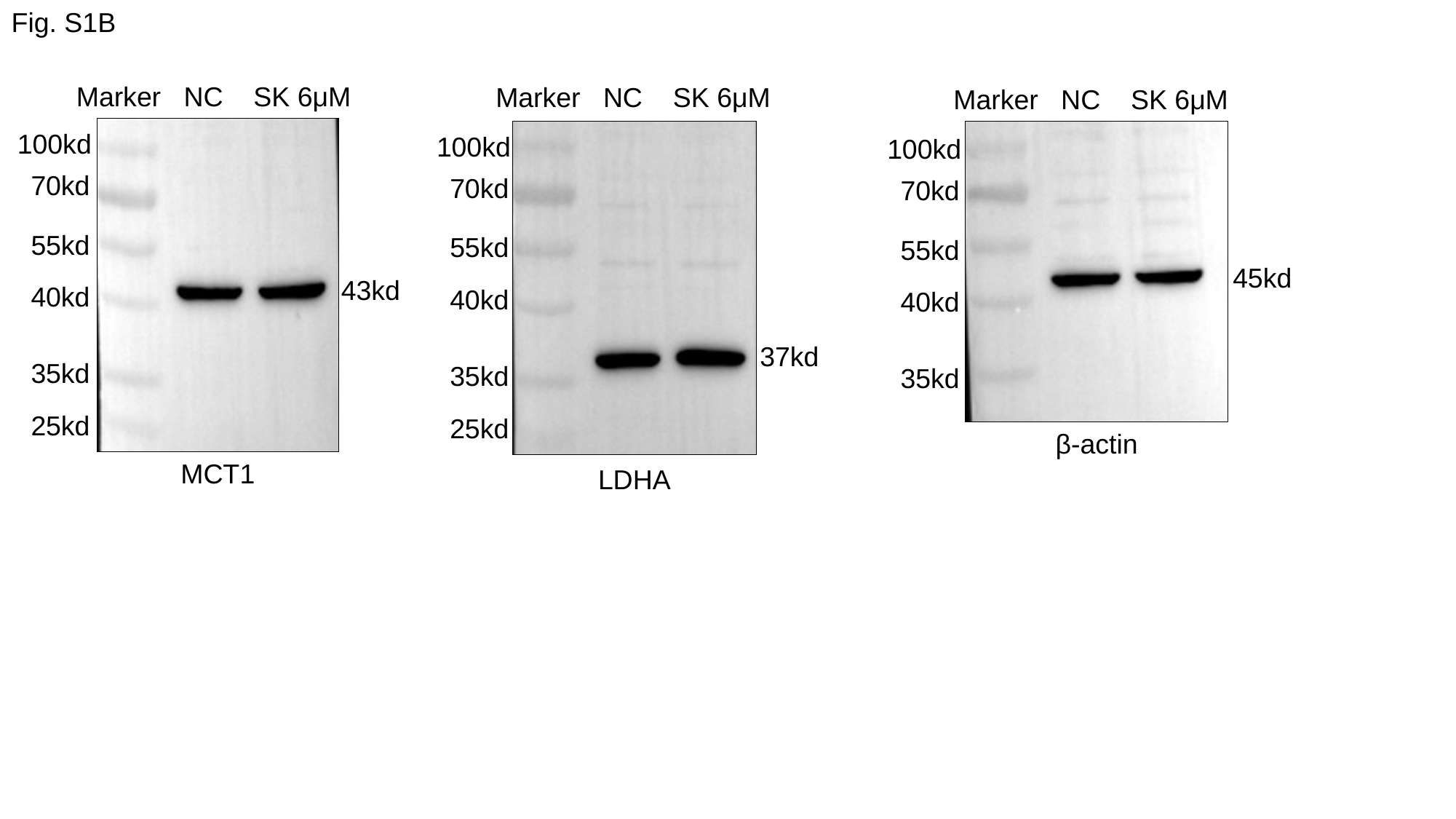

Fig. S1B
Marker NC SK 6μM
Marker NC SK 6μM
Marker NC SK 6μM
100kd
100kd
100kd
70kd
70kd
70kd
55kd
55kd
55kd
45kd
43kd
40kd
40kd
40kd
37kd
35kd
35kd
35kd
25kd
25kd
β-actin
MCT1
LDHA

## Slide 5
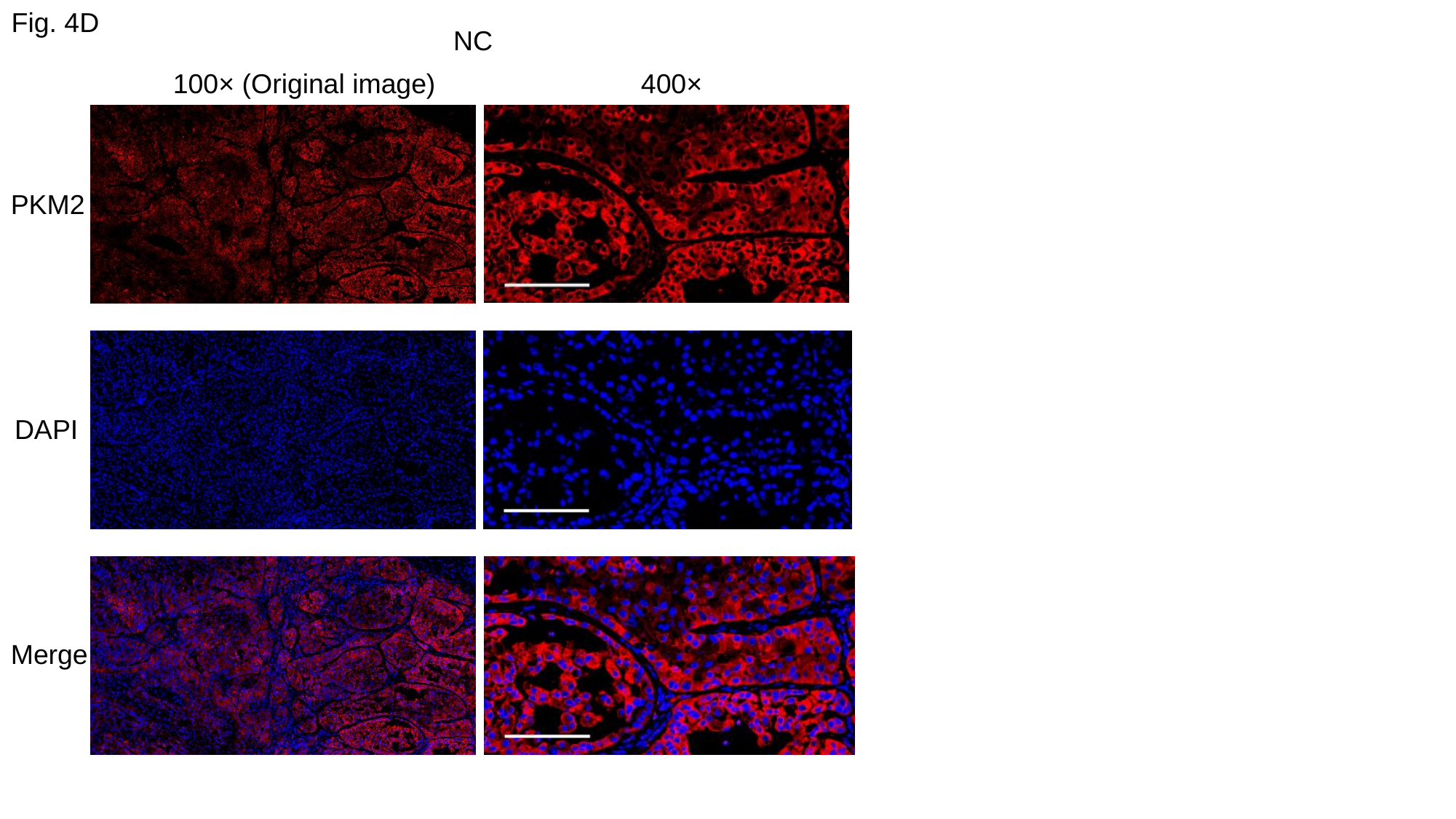

Fig. 4D
NC
100× (Original image) 400×
PKM2
DAPI
Merge

## Slide 6
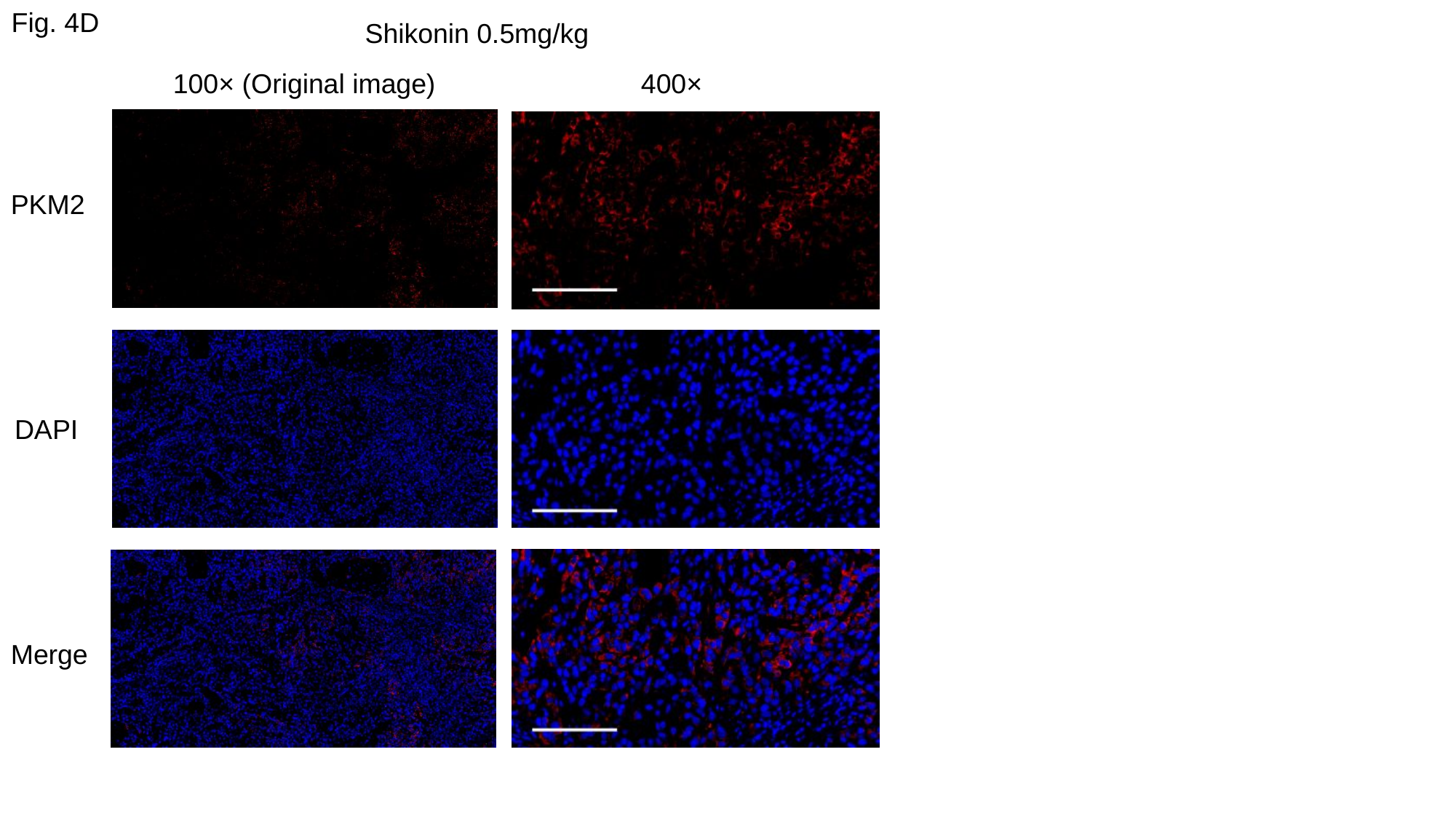

Fig. 4D
Shikonin 0.5mg/kg
100× (Original image) 400×
PKM2
DAPI
Merge

## Slide 7
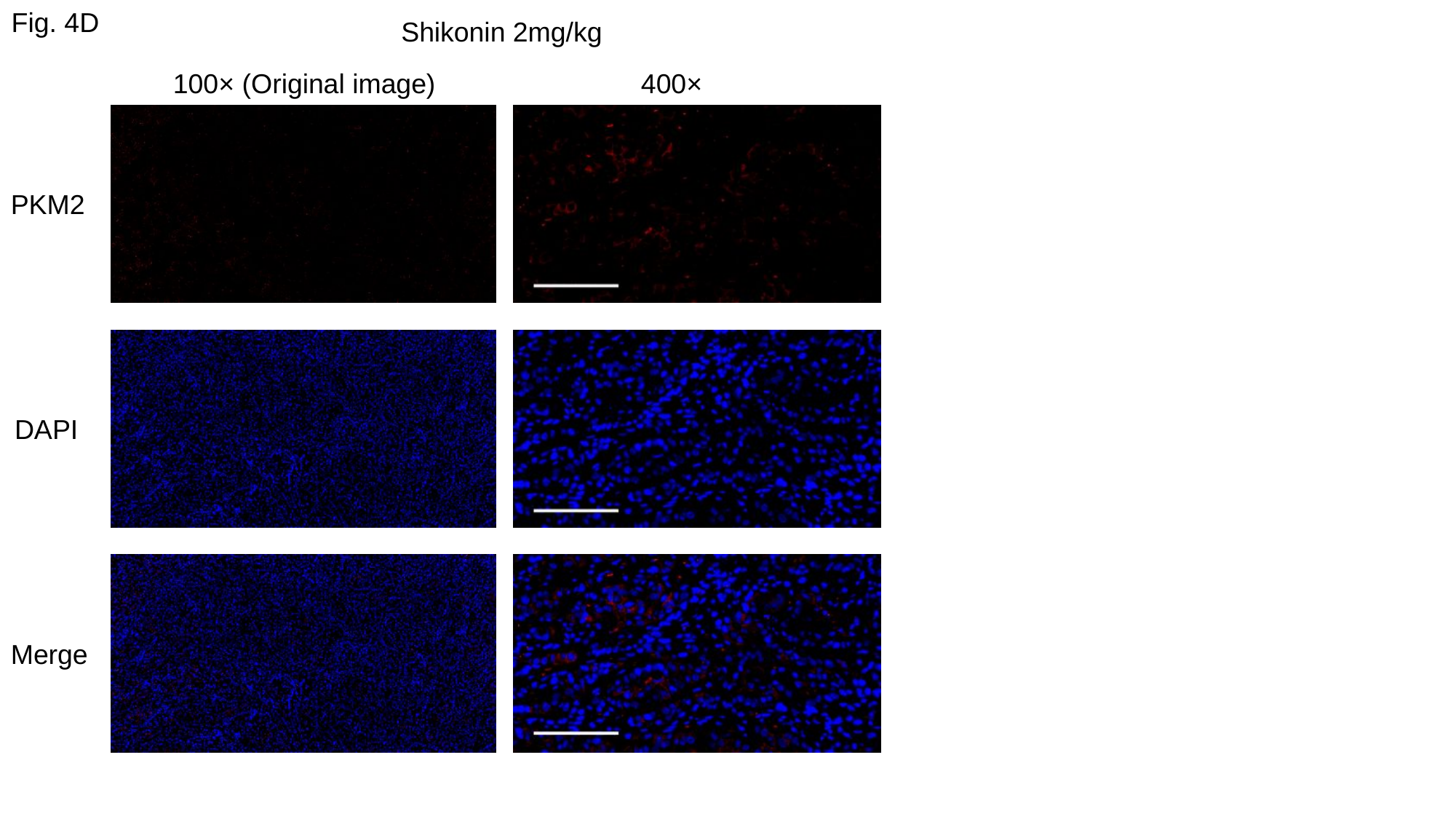

Fig. 4D
Shikonin 2mg/kg
100× (Original image) 400×
PKM2
DAPI
Merge

## Slide 8
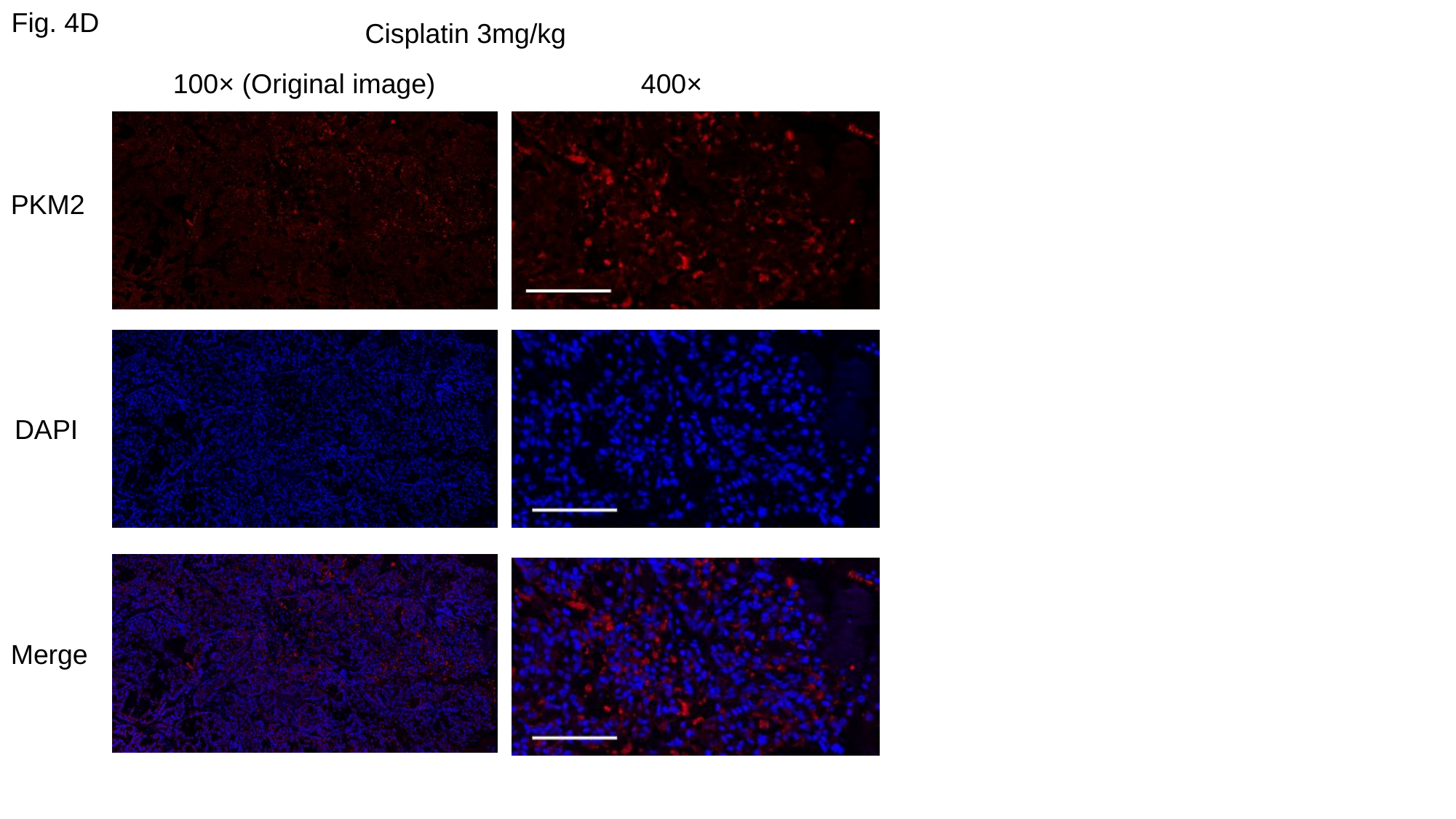

Fig. 4D
Cisplatin 3mg/kg
100× (Original image) 400×
PKM2
DAPI
Merge

## Slide 9
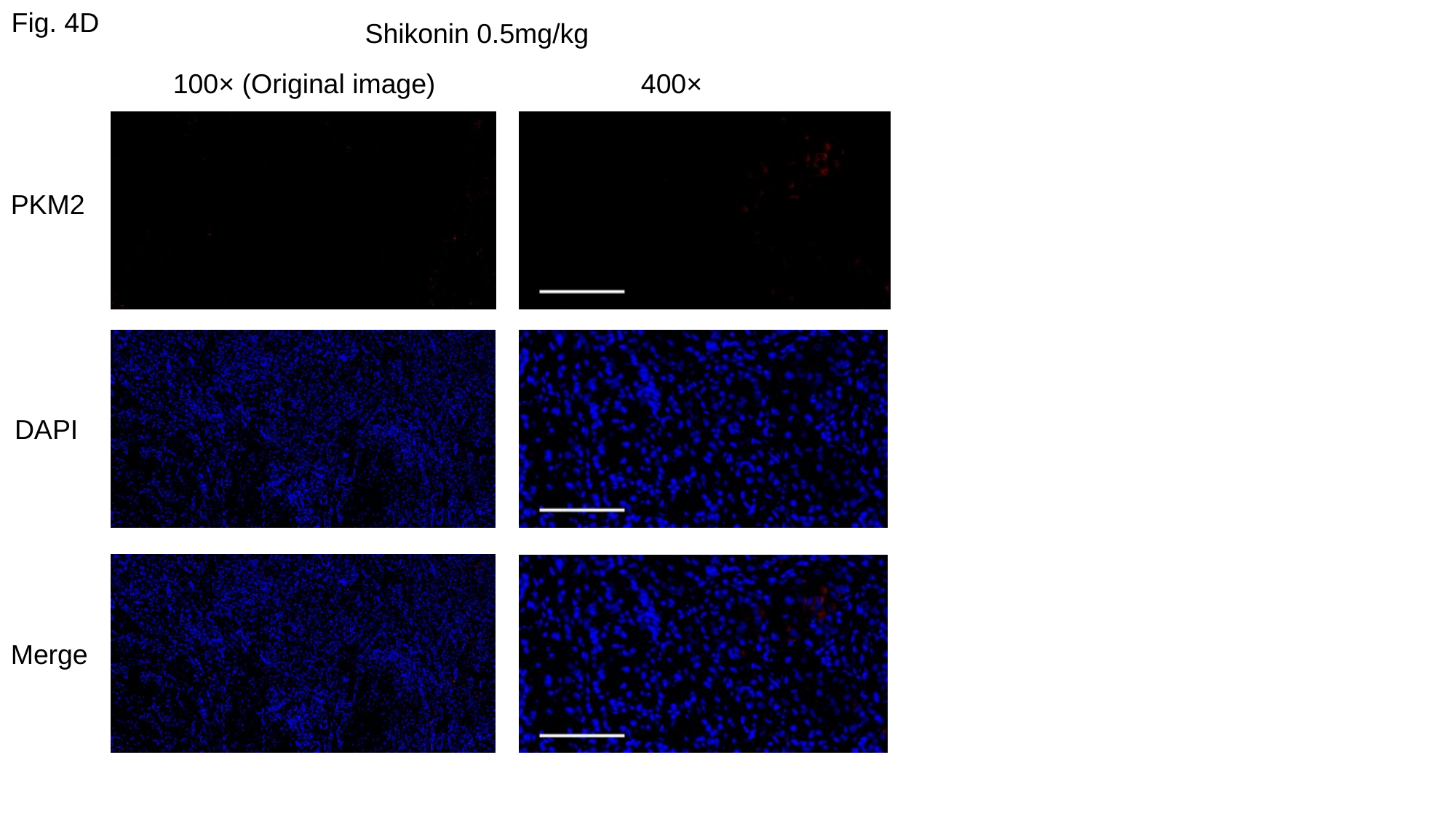

Fig. 4D
Shikonin 0.5mg/kg
100× (Original image) 400×
PKM2
DAPI
Merge
