# Supplementary material for: Shikonin inhibited glycolysis and sensitized cisplatin treatment in non-small cell lung cancer cells via the exosomal pyruvate kinase M2 pathway
Source: Bioengineered. 2022 Jun 15;13(5):13906–18. doi: 10.1080/21655979.2022.2086378 (PMC9275963; doi:10.1080/21655979.2022.2086378)
Supplement: Supplemental Material [file KBIE_A_2086378_SM9309.zip › supplementary/Supplementary Figure 1.docx]

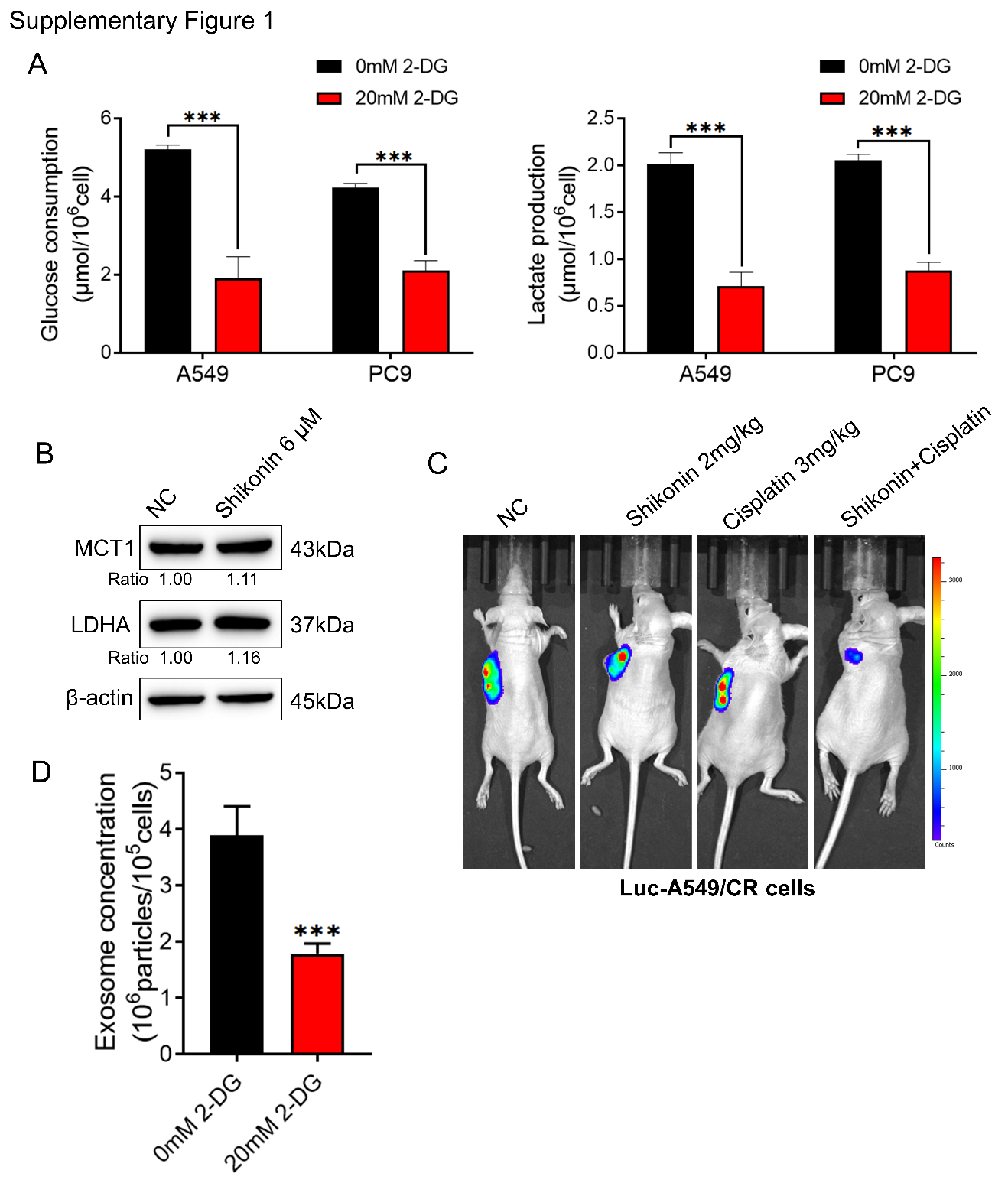


Supplementary Figure Legends

A. Glucose uptake and lactate production in A549 and PC9 cells after treatment with 20mM 2-DG.

B. Immunoblots and grayscale values of PKM2 expression in A549 cells treated with 20mM 2-DG.

C. Shikonin and cisplatin treatment were used in luciferase-labeled A549/CR cell-derived xenografts (CDX) tissue as our previous study *(Theranostics 2021; 11(6):2860-2875)*. 100 mm^3^ tumor tissue blocks were transplanted subcutaneously into mice, and drug treatment was started 2 days later, as described in Methods. Intravital luciferase imaging in mice was used to evaluate the combined treatment effect of shikonin and cisplatin. The results showed that the combination of shikonin and cisplatin significantly inhibited tumor growth in A549/CR cell-derived CDX models.

D. Relative concentrations of exosomes secreted by A549 cells were detected under treatment with 20mM 2-DG. ***p<0.001.
